# Superoxide dismutase down-regulation and the oxidative stress is required to initiate pupation in *Bombyx mori*

**DOI:** 10.1038/s41598-019-51163-3

**Published:** 2019-10-11

**Authors:** Yosui Nojima, Hidemasa Bono, Takeshi Yokoyama, Kikuo Iwabuchi, Ryoichi Sato, Katsuhiko Arai, Hiroko Tabunoki

**Affiliations:** 1 Department of United Graduate School of Agricultural Science, Tokyo University of Agriculture and Technology, 3-5-8 Saiwai-cho, Fuchu, Tokyo 183-8509 Japan; 2 Database Center for Life Science (DBCLS), Joint Support-Center for Data Science Research, Research Organization of Information and Systems (ROIS), Yata 1111, Mishima, Shizuoka, 411-8540 Japan; 3 Department of Science of Biological Production, Graduate School of Agriculture, Tokyo University of Agriculture and Technology, 3-5-8 Saiwai-cho, Fuchu, Tokyo 183-8509 Japan; 4 Bio-Applications and Systems Engineering, Tokyo University of Agriculture and Technology, Koganei, Tokyo 184-8588 Japan; 5 Department of Tissue Physiology, Tokyo University of Agriculture and Technology, Fuchu, Tokyo 183-8509 Japan

**Keywords:** Metalloproteins, Apoptosis

## Abstract

Perhaps, oxidative stress progresses pupation in some Lepidopteran insects; however, the reasons for this remain obscure. In our previous study, we clarified *Bombyx mori* SOD1 (BmSOD1) and *B. mori* SOD2 (BmSOD2) proteins respond in common to ultraviolet irradiation (UV) oxidative stress and metamorphosis. This result strongly suggested pupation initiates by oxidative stress and might mediate by down-regulation of expression of BmSOD1 and BmSOD2 proteins. Thus, we examined about these relationships in *B. mori* in this study. In the microarray data reanalysis, we found the Notch signaling pathways as the common pathways in pupation and UV oxidative stress in *B. mori*. Also, we showed a molting hormone, 20-hydroxyecdysone, leads not only generation of superoxide but also downregulation of the expression of BmSOD proteins during pupation in *B. mori*. Our findings can contribute to a deeper understanding of how biological defense systems work against environmental oxidative stress.

## Introduction

There are two major factors contributing to cellular stress in organisms. One comprises internal factors, such as respiration, production of energy, anti-inflammatory, and redox system. The other comprises external factors, such as environmental factors, ultraviolet (UV) irradiation, infection, temperature, and humidity^1^. These stress factors can induce the generation of reactive oxygen species (ROS), which are constantly produced in eukaryotes and prokaryotes^2^. Some common reactive oxygen derivatives are the superoxide anion radical (O_2_^−^), hydrogen peroxide (H_2_O_2_), the hydroxyl radical (OH^−^), the nitric oxide radical (NO^−^), and peroxynitrite (ONOO^−^). These ROS cause oxidative damage to proteins, oxidation of lipids, and DNA damage in the cell and are, therefore, toxic to living organisms. However, organisms have systems for controlling levels of ROS in their cells.

Superoxide dismutase (SOD) is a metalloprotein that scavenges O_2_^−^and converts it into hydrogen peroxide^3^. Three kinds of SOD are present in eukaryotes. SOD1 and SOD3 bind with copper and zinc ions in their active sites and SOD2 binds with manganese ions. SOD1 is mainly localized in the cytosol, SOD2 is localized in the mitochondria, and SOD3 is secreted into the extracellular space^4^. Thus, they each play distinct roles in the cell.

*Bombyx mori* has been utilized as an agricultural model insect because its genome is well-characterized and compatible with microarray technologies^5,6^. *B. mori* has a much larger body size than *Drosophila melanogaster* and the function of its proteins can be analyzed using each tissue. The three types of SOD proteins described above (SOD1, SOD2, and SOD3) have also been found in *B. mori*^7–9^. In addition, four type of other *B. mori* SOD gene have been found^10^. In a previous study, we clarified that *B. mori* SOD1 (BmSOD1) and *B. mori* SOD2 (BmSOD2) proteins responded to UV irradiation and metamorphosis in the fat body of the fifth instar larva^11^. This result strongly suggested pupation initiates by oxidative stress and might mediate by down-regulation of expression of BmSOD1 and BmSOD2 proteins.

Furthermore, several insect studies have correlated the generation of ROS resulting from environmental oxidative stress with developmental processes. For example, hypoxia stress was found to promote the action of wandering during pupation in the tobacco hornworm (*Manduca sexta*)^12^. In another case, the administration of isosorbide dinitrate, a NO donor, to the beetle *Homoderus mellyi Parry* rapidly advanced the process of pupation^13^. Thus, the generation of ROS in response to environmental oxidative stress appears to be closely related to the initiation of developmental events in insects; however, the molecular mechanisms why oxidative stress progresses metamorphosis remains unclear. In this study, we examined the relationship between internal and external stresses and the generation of superoxide which one of the ROS in *B. mori*. And we found superoxide utilized for pupation by down-regulated expression of BmSOD1 and BmSOD2 proteins.

## Results

### UV irradiation and pupation share a common molecular pathway in *B. mori*

**Figure 1:**
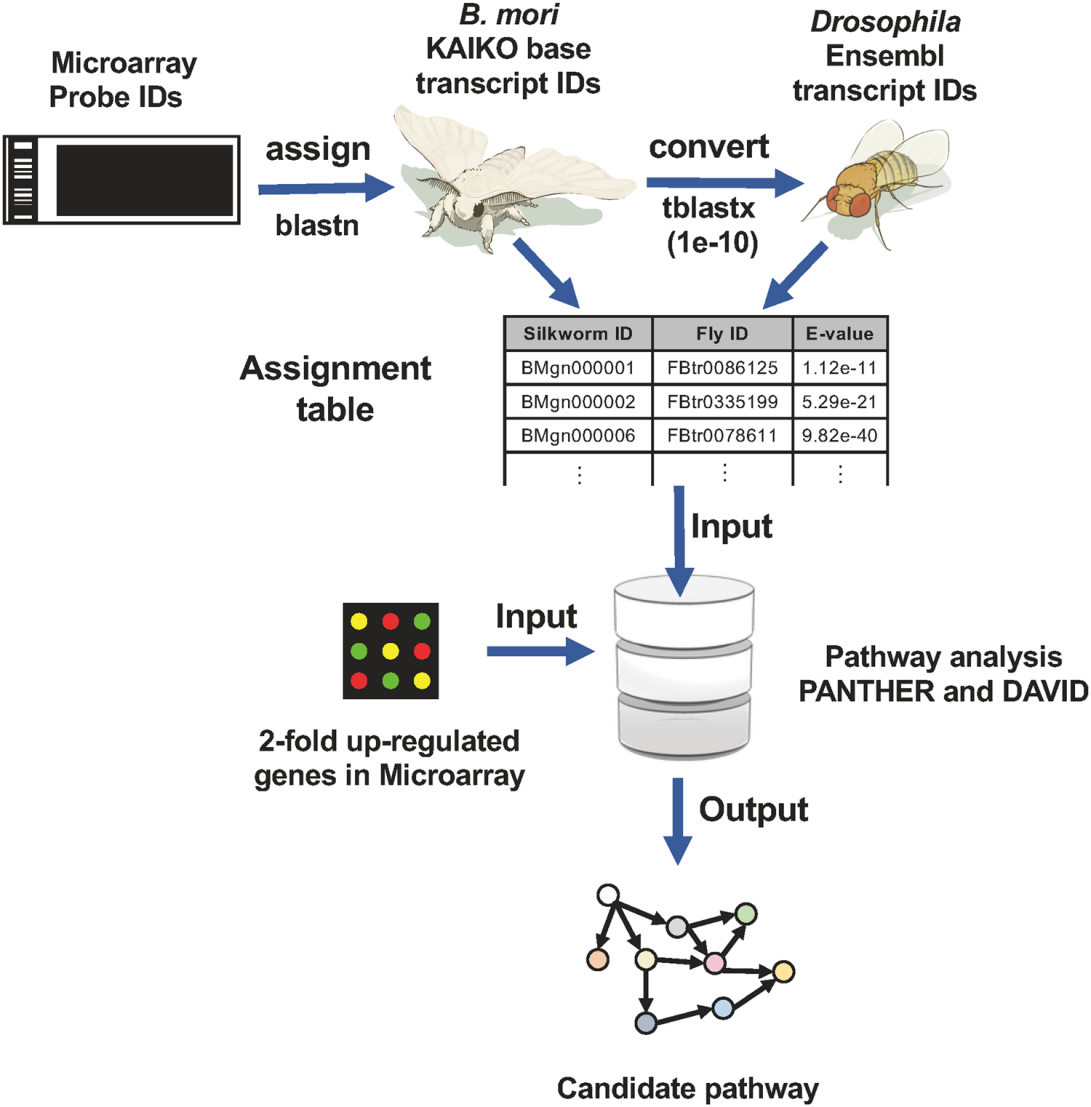
Construction of a gene assignment pipeline for KAIKO microarray datasets. To analyze KAIKO microarray datasets, we constructed a gene assignment pipeline from KAIKO gene IDs to fly gene IDs for conversion of two-fold upregulated gene IDs from GSE55816 and GSE23424. Converted gene IDs were applied to pathway analysis using PANTHER and DAVID (cut-off threshold; count ≥2 and *P* < 0.05). Ms. Mai Sakamoto from Tabunoki laboratory gifted the fly and silkworm image drawings. Nojima Y and Tabunoki H made microarray slide, signal, conversion table and pathway image drawings. The database image drawing (http://g86.dbcls.jp/~togoriv/) are licensed under the HYPERLINK http://creativecommons.org/licenses/by/4.0/deed.ja Creative Commons display 4.0 license http://creativecommons.org/licenses/by/4.0/deed.ja.

Environmental oxidative stress induces metamorphosis in insects^12,13^. Thus, we hypothesized that insect molecular signaling pathways are similar in the internal and external oxidative stresses. To investigate which molecular signaling pathways might be common in the internal and external oxidative stresses, we used two microarray data sets GSE55816 and GSE23424 from the public database NCBI Gene Expression Omnibus (GEO). The GSE55816 dataset contains gene expression data from the *B. mori* fat body irradiated with UV at 29.16 and 58.32 J/cm^2^. The GSE23424 dataset contains gene expression data from the fat bodies of fourth molting (4M), fifth feeding (5F), and pre-pupa (PP) of *B. mori*. A summary of the microarray analysis pipeline is shown in Fig. 1 (see Methods for details). We identified the Notch signaling pathway as a candidate pathway shared among UV irradiation at 58.32 J/cm^2^, fourth molting stage (4M/5F), and prepupal stage (PP/5F) (Tables 1 and 2). We also identified the Hippo and TGF-beta signaling pathways as common pathways between 4M/5F and PP/5F stages (Table 1).

**Table 1:** Gene sets enrichment analysis of upregulated genes in fourth instar larva on molting and pre-pupa.

ID	PANTHER Pathways	Count	P-value
**4M/5F (fourth instar larva on molting)**			
P00045	Notch signaling pathway	4	1.43E-02
P00034	Integrin signaling pathway	5	4.41E-02
**ID**	**KEGG pathways**	**Count**	**P-value**
dme04391	Hippo signaling pathway in fly	9	6.53E-03
dme04512	ECM-receptor interaction	4	1.43E-02
dme04330	Notch signaling pathway	5	2.03E-02
dme00564	Glycerophospholipid metabolism	8	2.36E-02
dme04350	TGF-beta signaling pathway	6	2.92E-02
**PP/5F (pre-pupa)**			
**ID**	**PANTHER Pathways**	**Count**	**P-value**
P00045	Notch signaling pathway	5	3.42E-03
**ID**	**KEGG Pathways**	**Count**	**P-value**
dme04330	Notch signaling pathway	7	8.25E-04
dme04391	Hippo signaling pathway in fly	11	8.82E-04
dme04144	Endocytosis	16	1.67E-03
dme04142	Lysosome	12	1.05E-02
dme04350	TGF-beta signaling pathway	6	4.22E-02

**Table 2:** Gene sets enrichment analysis of upregulated genes after UV irradiation.

ID	PANTHER Pathways	Count	P-value
**29.16 J/cm** ^**2**^ **_upregulated**			
P00014	Cholesterol biosynthesis	2	3.39E-03
P00059	P53 pathway	2	3.16E-02
P00023	General transcription regulation	2	4.34E-02
**ID**	**KEGG Pathways**	**Count**	**P-value**
dme04141	Protein processing in endoplasmic reticulum	12	4.26E-04
**58.32 J/cm** ^**2**^ **_upregulated**			
**ID**	**PANTHER Pathways**	**genes**	**P-value**
P00045	Notch signaling pathway	3	1.26E-02
P00023	General transcription regulation	3	1.81E-02
P00055	Transcription regulation by bZIP transcription factor	3	4.15E-02
**ID**	**KEGG Pathways**	**genes**	**P-value**
No hits found			

### Identification of expression patterns of BmSOD1 and BmSOD2 proteins during the developmental process by immunoblotting

**Figure 2:**
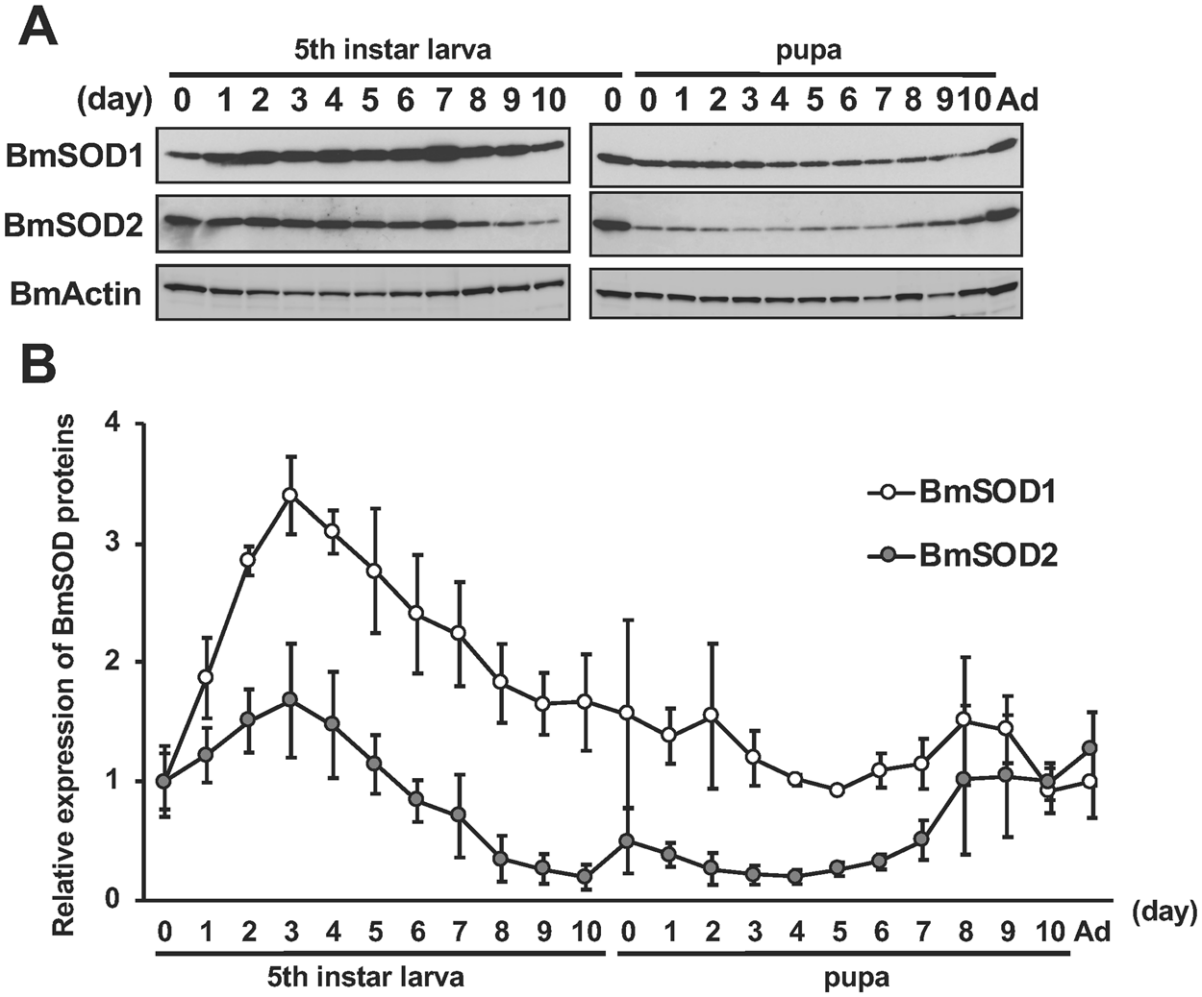
Developmental distribution of the expression of both BmSOD proteins in the fat body. **(A)** Expression of BmSOD1 and BmSOD2 proteins in the fat body from day 0 of fifth instar larva to the adult stage. Aliquots (10 µg) of the fat body lysate were separated by 15% SDS-PAGE; transferred to nitrocellulose; and probed with BmSOD1, BmSOD2, or actin antibodies. BmActin was used as an endogenous control. (B) Relative expression levels (mean ± SD, n = 12) of both BmSOD proteins, normalized to the expression of BmActin protein. The relative expression levels were calculated using day 0 of the fifth instar larvae sample as 1. Ad indicates day 0 of adult.

To examine the expression of BmSOD1 and BmSOD2 proteins in *B. mori* cells and tissues, we prepared antiserums against the recombinant X-press-tagged BmSOD1 and BmSOD2 to identify BmSOD1 and BmSOD2 proteins (Supplementary Fig. S1A). In addition, we examined the effectiveness of a commercial anti-actin antibody raised against the C-terminus of human actin to identify *B. mori* actin (BmActin) (Supplementary Fig. S1B). We confirmed the specificity of these antibodies and concluded that these antiserums and antibodies would be useful for detecting objective proteins. We investigated the expression of BmSOD1 and BmSOD2 in the fat body during the fifth instar larval stage to the adult stage and determined that the expression of both proteins gradually decreased from day 3 of fifth instar larval to early pupal stages (Fig. 2A,B). The expression of both BmSOD proteins markedly fluctuated during late larval and pupal developmental process.

### Measurement of the amount of superoxide produced during the developmental process by dihydroethidium (DHE) staining

**Figure 3:**
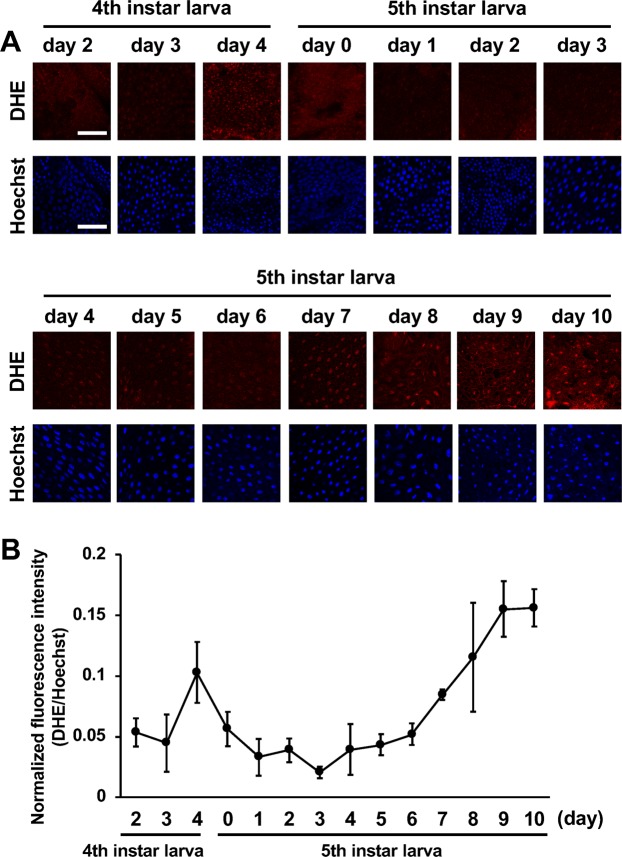
Production of superoxide in the fat body during fourth instar to wandering stage. **(A)** DHE and Hoechst stained fat body from day 2 of fourth instar larva to day 10 of fifth instar larvae. The scale bar indicates 80 µm. **(B)** Quantification of the fluorescence intensity on DHE staining. DHE fluorescence intensity normalized by Hoechst fluorescence intensity and plotted on the graph as mean ± SD (n = 3).

To examine the presence of superoxide which is one of a ROS, particularly the superoxide anion, we measured the amount of superoxide in the fat body during the fourth to late fifth instar larva using DHE staining. The amount of superoxide increased at day 4 of the fourth instar, then gradually increased from day 7 to 10 of the fifth instar larva (Fig. 3A,B).

### Effect of 20E on the expression of both BmSOD proteins in the fat body

**Figure 4:**
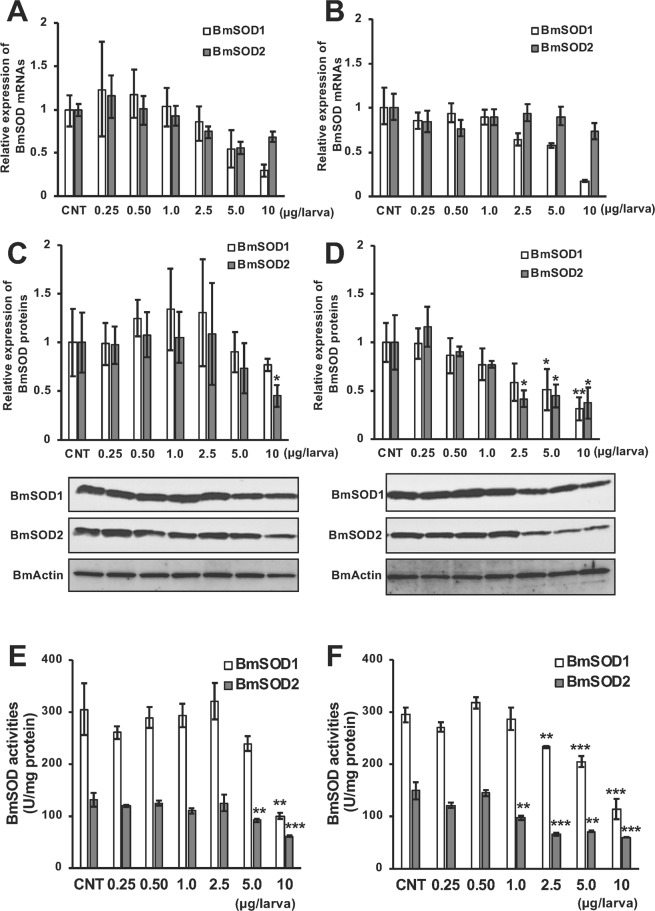
mRNA and protein expression of BmSOD1 and BmSOD2 and both SOD activity in the fat body after 20E treatment. Fat bodies were dissected from *B. mori* larvae and treated with 0-, 0.25-, 0.50-, 1.0-, 2.5-, 5.0-, or 10-µg/larva 20E after 24 or 48 h. Zero microgram/larva indicates control (CNT) injected with only 10% isopropanol. mRNA expression of BmSOD1 and BmSOD2 examined by qRT-PCR after 24 **(A)** or 48 h **(B**) and plotted as relative quantification (RQ) value compared with CNT. Error bars indicate relative minimum/maximum expression levels against mean RQ values. 18 s rRNA was used as the endogenous control. Expression of the BmSOD1 and BmSOD2 proteins were examined by immunoblotting after 24 **(C)** or 48 h (**D)**. Aliquots (5 µg) of the fat body lysate samples were separated on a 15% SDS-PAGE gel and immunoblotted. The BmActin protein was used as an endogenous control. The bands intensity of BmSOD1 and BmSOD2 proteins were calculated by image J and then, that normalized with the band intensity of BmActin protein and plotted as relative expression levels compared with CNT. Error bars indicate SD (n = 12). SOD activities were measured after 24 **(E)** or 48 h **(F)**. Error bars indicate SD (n = 12). *P < 0.05; **P < 0.01; and ***P < 0.001.

Because ecdysone plays an important role for pupation in *B. mori*, we assessed the relationship between ecdysone and expression of BmSOD to examine the effect of increasing the amount of superoxide in the fat body. 20E was injected into day 4 of fifth instar larvae, and the fat bodies were dissected after 24 or 48 h. For BmSOD1, expression of mRNA and protein were significantly decreased in 10 µg/larva injection of 20E after both 24 and 48 h (Fig. 4A–D). While, expression of BmSOD2 mRNA was decreased in 10 µg/larva injection of 20E after both 24 and 48 h (Fig. 4A,B), and expression of BmSOD2 protein were significantly decreased in 10 µg/larva injection of 20E after both 24 and 48 h (Fig. 4C,D). In addition, SOD activities were markedly decreased for both BmSODs after 10 µg/larva injection of 20E after both 24 and 48 h (Fig. 4E,F).

### Effect of 20E on increasing amount of superoxide in the fat body

**Figure 5:**
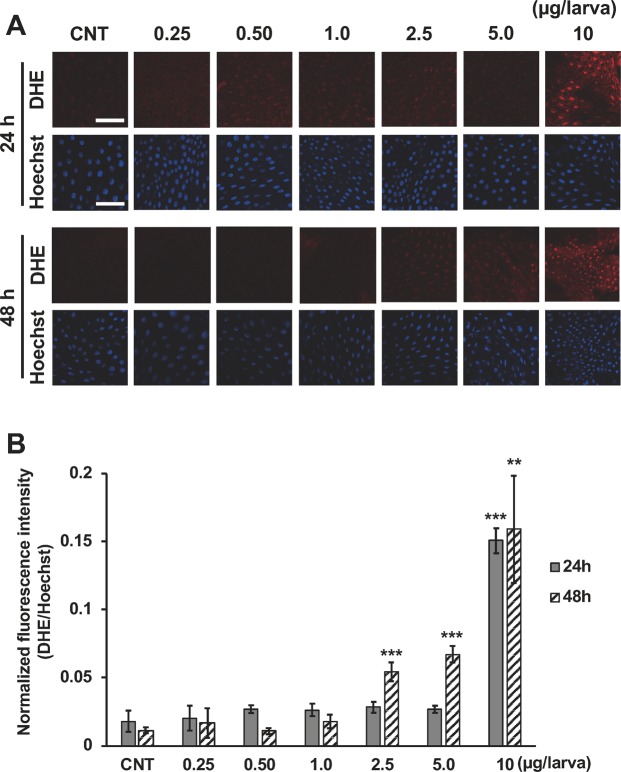
Production of superoxide in the fat body after 20E treatment. Fat bodies were dissected from *B. mori* larvae treated with 0-, 0.25-, 0.50-, 1.0-, 2.5-, 5.0-, or 10-µg/larva 20E after 24 or 48 h. Zero microgram/larva indicates control (CNT) injected with only 10% isopropanol. **(A)** Fat body stained with DHE and Hoechst. The scale bar equals 80 µm. **(B)** Quantification of fluorescence intensity on DHE staining. DHE fluorescence intensity normalized by Hoechst fluorescence intensity and shown as mean ± SD (n = 3). **P < 0.01 and ***P < 0.001.

We also examined the relationship between superoxide and ecdysone and found that, the amount of superoxide was increased with 10 µg/larva treatment after 24 h compared with that in the control group (Fig. 5A,B). Furthermore, the amount of superoxide was increased with the 2.5, 5.0, and 10 µg/larva treatments after 48 h compared with that in the control group (Fig. 5A,B).

### 20E induced mRNA expression of Notch signaling pathway- and programmed cell death (PCD)-related genes in the fat body

**Figure 6:**
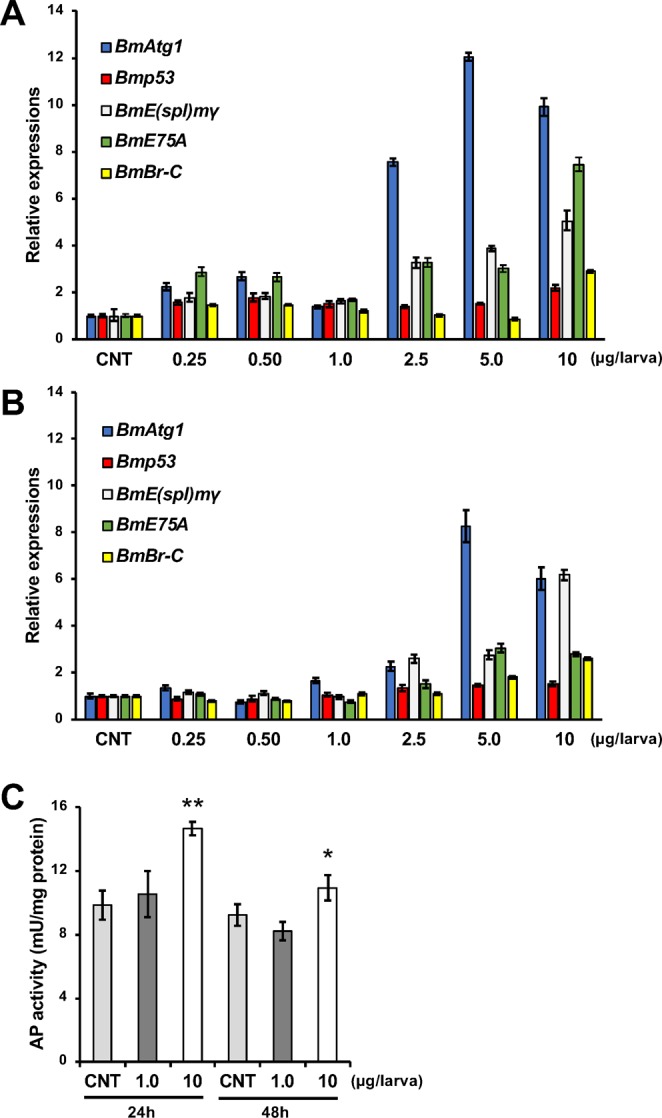
Expression of programmed cell death-related and Notch signaling pathway-related genes after 20E treatment in fat bodies of *B. mori* larvae. Fat bodies were dissected from *B. mori* larvae treated with 0-, 0.25-, 0.50-, 1.0-, 2.5-, 5.0-, or 10-µg/larva 20E after 24 or 48 h. Zero microgram/larva indicates control (CNT) injected with only 10% isopropanol. mRNA expression of BmAtg1, Bmp53, BmE(spl)mγ, BmE75A, and BmBr-C examined by qRT-PCR after 24 **(A)** or 48 h (**B**) are plotted as relative quantification (RQ) value compared with CNT. Error bars indicate relative minimum/maximum expression levels against mean RQ values. 18 s rRNA was used as the endogenous control. (**C**) Acid phosphatase (AP) activity measured in the fat body after 0-, 1.0-, or 10-µg/larva 20E treatment after 24 or 48 h. Error bars indicate SD (n = 3). *P < 0.05 and **P < 0.01.

We investigated whether the Notch signaling or PCD pathway were induced in the fat body of *B. mori* larvae by 20E injection. *BmAtg1* and acid phosphatase (AP) activity are related to autophagy^14^, *Bmp53* is related to apoptosis^15^, and *BmE(spl)mγ* is related to the Notch signaling pathway^16^. All of these were examined in the fat body of 20E-injected larva by qRT-PCR. mRNA expression of *BmAtg1* was dramatically increased with 5.0 and 10 µg/larva treatments after both 24 and 48 h, and AP activity was increased with 10 µg/larva treatment after 24 and 48 h (Fig. 6A–C). mRNA expression of *Bmp53* was increased with 10 µg/larva treatment after 24 h (Fig. 6A). mRNA expression of *BmE(spl)mγ* was also increased with 10 µg/larva treatment after both 24 and 48 h (Fig. 6A,B). We confirmed that the expression of *BmE75A* and *BmBr-C*, which are known as 20E responsive genes^17,18^, were markedly increased with 20E injection (Fig. 6A,B).

### SOD-like chemical injection into *B. mori* larvae

Finally, we examined the relationship between the expression of BmSODs and pupation. We employed an SOD-like chemical (SOD mimic) for increasing the potency of SOD in the larval body of *B. mori*. The *B. mori* strain Kinsu x Showa used in this study pupates on day 10 of the fifth instar larva.

**Figure 7:**
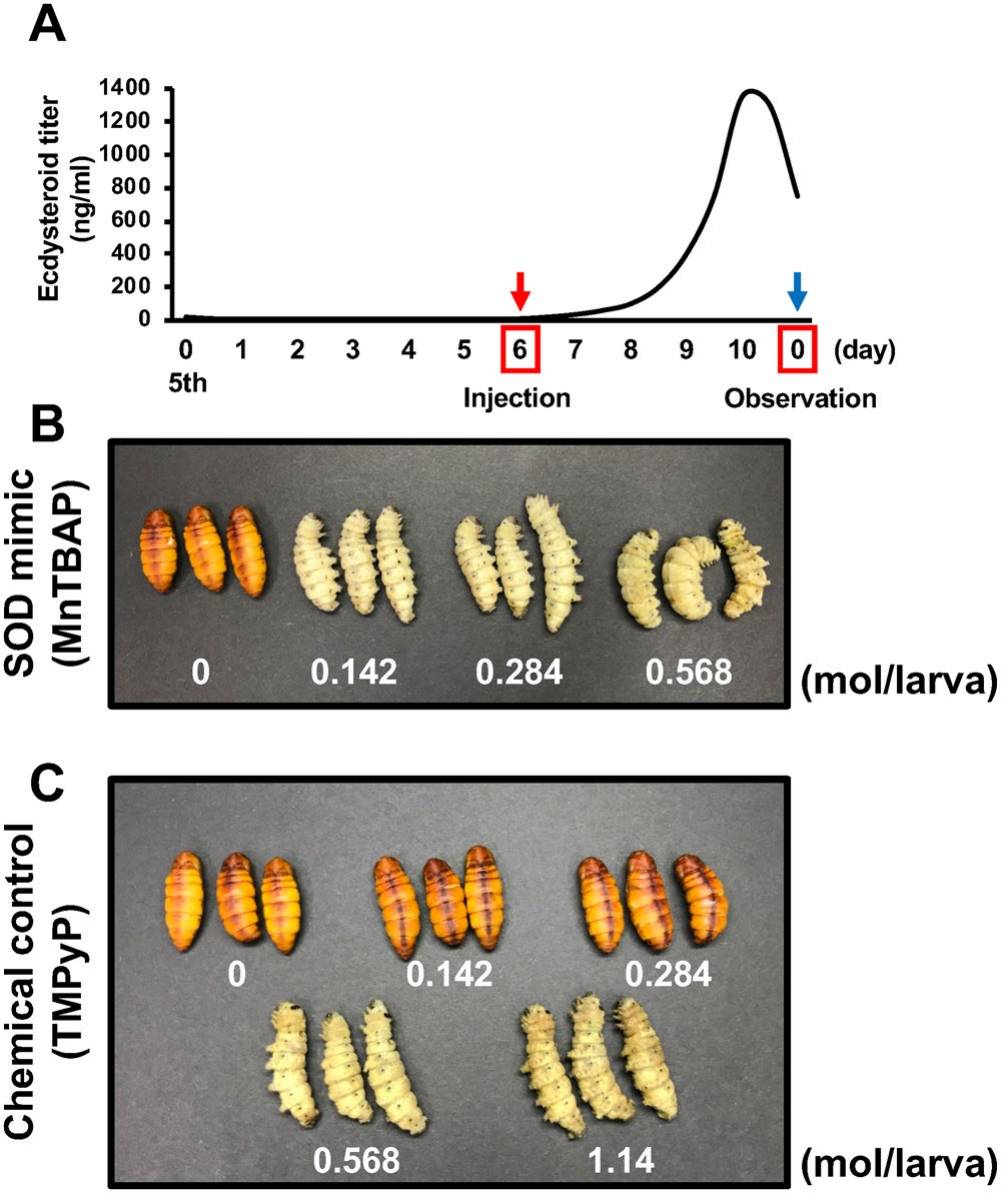
SOD mimic suppressed pupation in *B. mori* larvae. **(A)** The ecdysteroid titer in the hemolymph of the fifth instar larva was modified as described by Kamimura *et al*.^33^. The day on which the SOD mimic or TMPyP was injected is shown with a red arrow; day of observation and pupation is indicated with the blue arrow. **(B)** SOD mimic was injected into day 6 of fifth instar larva at concentration of 0, 0.142, 0.284, or 0.568 mol/larva (p = 0.0006). TMPyP was injected into day 6 of fifth instar larva at concentration of 0, 0.142, 0.284, 0.568, or 1.14 mol/larva. The image shows injected larvae as observed after 5 days.

Thus, the potency of SODs were increased using the SOD mimic manganese (III) tetrakis (4-benzoic acid) porphyrin chloride (MnTBAP) on the day of fifth instar larva (Fig. 7A). MnTBAP is known as a porphyrin that has an SOD-like activity^19,20^. 5,10,15,20-tetrakis(1-methyl-4-pyridinio)porphyrin tetra(*p*-toluenesulfonate) (TMPyP) is also known as a porphyrin, but it does not have the potency of a SOD mimic^21^. At 5 days after injection, *B. mori* larvae injected with 0.142–0.568 mol/larva of MnTBAP were not pupated until the period of pupation (Fig. 7B); however, *B. mori* larvae injected with 0.142 and 0.284 mol/larva of TMPyP were pupated until the period of pupation (Fig. 7C). Thus, pupation was somewhat inhibited by SOD mimic injection.

## Discussion

In this study, we examined the relationship among the expression of BmSOD1 and BmSOD2 proteins, UV oxidative stress and pupation in *B. mori*, and investigated how two BmSOD proteins works to control the generation of superoxide which one of ROS. First, we searched for pathways common to both pupation and UV oxidative stress from publicly available data in NCBI GEO, identifying the Notch signaling pathways as shared pathways. We also identified the Hippo and TGF-beta signaling pathways as common pathways in the fourth molting stage and the pupal stages. The Notch signaling pathway is involved not only in the development^22–24^ but also in controlling the generation of ROS^25–27^. The Hippo signaling pathway induces apoptosis in the insect^28^, also apoptosis is induced in the fat body of the *B. mori* pre-pupa^29,30^. The TGF-beta/activin signaling pathway is involved in the production of ecdysteroid^31,32^. We found that even though these molecular pathways were induced by different factors, and Notch signaling pathway was common to both internal and external stress.

While, we found the BmSOD1 protein in the cytosol, the BmSOD2 protein was present in the mitochondria (Supplementary Fig. S2). The expression of both BmSOD1 and BmSOD2 was decreased in the fat body from day 7 of the fifth instar larva to day 5 of the pupa (Fig. 2A,B). In addition, the expression of BmSOD2 was decreased in the Malpighian tubule and midgut during the early pupal stage (Supplementary Fig. S3). Conversely, the amount of superoxide gradually increased in the fat body from day 7 of the fifth instar larva (Fig. 3A,B). This period constitutes the wandering stage of the larvae that occurs before pupation corresponding to an increase in the secretion of ecdysteroids into the hemolymph^33,34^. Therefore, an increased amount of superoxide was correlated with an increase in the ecdysteroid titer in the hemolymph.

We also assessed the expression of both BmSOD proteins in the fat body during the fourth larval developmental stage and determined that the expression of both BmSOD proteins tended to decline on day 3 of fourth instar larva, and then these expressions turned back before moulting (Supplementary Fig. S4) in the fourth instar larval developmental stage. Kamimura *et al*. reported that the ecdysteroid titre is the highest on day 3 of the fourth instar larva, following which the ecdysone titre decreases after day 4 of the fourth instar larva^33^. Previously, we predicted that both BmSOD proteins could be worked in removing superoxide, which is induced by ecdysone in the moulting event because both BmSODs expressions were also induced by UV irradiation oxidative stress^11^. Hence, perhaps, the amount of superoxide might be controlled by the ecdysone titre and both BmSOD proteins might work for removing superoxide.

After assessing the effect of 20E on BmSOD1 and BmSOD2, we established that the function of both BmSODs was downregulated with the 20E treatment, whereas the amount of superoxide was markedly increased. In our experiment, *B. mori* larvae responded to 20E, which is 10 times higher than the physiological condition^33^. Although we injected the physiological level of 20E on day 4 of fifth instar larvae, *B. mori* larvae marginally responded to the physiological concentration of 20E in this study, suggesting that 20E could be mandatorily downregulated by their physiological system on day 4 of fifth instar larvae. As this period displayed very low level of 20E in the larval developmental stage, we needed more high concentration of 20E in these experiments. Another study reported the administration of 20E dose of 10 µg/larva to the fourth instar larva^35^. In addition, 20E was administered at 5 µg/larva on day 2 of the fifth instar larva to p50 strain of *B. mori*^14^. Kinshu × Showa have about three to four times of big body size than p50 (Supplementary Fig. S5). Thus, it was considered that the 20E dose is experimentally appropriate in this study; these experiments indicate that 20E is crucial for the control and generation of superoxide and the modulation of the expression of BmSOD proteins as a trigger during the late larval to early pupal developmental stages. Reportedly, superoxide is primarily produced in the mitochondrial electric transport system^1,36^; our findings further suggest that the BmSOD2 protein is of particular importance for removing superoxide in the *B. mori* body when pupation.

Decreases in the expression of the insect SOD2 correlate with developmental events in other insect species. For example, mRNA expression of TcSOD2 was found to be significantly decreased in the pharate pupa of the red flour beetle *Tribolium castaneum*^37^, whereas mRNA expression of AcSOD2 was also found to be decreased from the late larval to the early pupal stages of the eastern honey bee *Apis cerana cerana*^38^. Accordingly, decreased expression of SOD2 should be associated with pupation in other species, and our findings further support that this relationship might be conserved across holometabolous insects.

Even though BmSOD1 and BmSOD2 are important superoxide scavengers in *B*. *mori*, the expression levels of both BmSOD proteins should decrease during pupation to enable an increase in the generation of superoxide. Pupation is initiated from the late larval to prepupal developmental stages in *B. mori*. Autophagy and apoptosis are induced in the fourth molting stage and prepupal periods for tissue remodeling of the *B. mori* larvae^14,30^ and continue during the early pupal developmental period as dynamic remodeling of the body structure occurs^13^. Pupation is initiated at the late larval stage when the concentration of ecdysteroids increases in the hemolymph^33^. The neuropeptide prothoracicotropic hormone (PTTH) levels increase under specific physiological stimuli to produce and release ecdysteroids from the prothoracic gland^34^. ROS would be then produced through the ecdysteroid signaling pathway that is stimulated by secretion of PTTH.

Our results show that expression levels of both BmSODs decreased from the late larval to the pupal development stages, coinciding with the initiation of autophagy. In fact, we found that mRNA expression of *BmAtg1* (an autophagy-related gene) and AP activity were significantly increased in the fat body with 20E treatment. The expression of BmAtg1 mRNA was initiated in the late larval developmental stage to the early pupal developmental stage; this span correlated with tissue remodelling for pupation (Supplementary Fig. S7). In addition, pupation was slightly inhibited by a SOD mimic injection into larvae (p = 0.0006). Although they could make a cocoon, they could not moult for pupation (Supplementary Fig. S6). We observed after 3 days and counted the dead individuals, which were observed in the 0.142 and 0.284 mol/larva SOD mimic treatment groups, but no dead individuals were observed in the 0.568 mol/larva SOD mimic treatment group after 3 days (Supplementary Table S2). In this experiment, it remains unclear whether the individual died because of SOD mimic toxicity. SOD mimic is not harmful to the mammalian model, and the lethal dose of 50% (LD50) and toxicological information do not exhibit in the safety data sheet^39^. Hence, the SOD mimic seemed to be inhibiting pupation. These results suggest that the expression of BmSODs should be controlled by increases in autophagy from superoxide when restructuring the *B. mori* larval tissue because pupation might be initiated by increasing superoxide.

Furthermore, our findings indicate that *BmE(spl)mγ* mRNA, a downstream gene of the Notch signaling pathway^16^, is increased in the fat body under 20E treatment. The Notch signaling pathway plays a role in modulating the amount of ROS in organisms^25–27^, and thus, pupation could be controlled by the balance between the generation of superoxide and the expression of SODs. In future studies, we intend to investigate those factors controlling the expression of BmSOD1 and BmSOD2 by ecdysteroids. Furthermore, we aim to introduce superoxide treatment with a suitable compound to fifth instar larvae and observe whether those larvae pupate as quickly as usual.

In conclusion, we examined relationships among BmSOD1, BmSOD2, and pupation in *B*. *mori*, finding that their expression patterns were inversely correlated with ecdysone levels during the prepupal stage, along with the amount of superoxide in the fat body cells. These findings indicate that the BmSOD1 and BmSOD2 proteins commonly play a role in the removal of superoxide produced in response to internal and external oxidative stress. Our study shows that the physiological generation of ROS leads to the initiation of pupation and that ecdysone plays a role in controlling the generation of ROS and down-regulating the function of BmSODs in *B*. *mori*. Our findings will contribute to an increased understanding of biological defence systems against environmental oxidative stress, such as UV irradiation, and the development of the holometabolous insect.

## Methods

### Insects

The *B. mori* hybrid strain Kinshu x Showa, supplied by Ueda-Sha Co. Ltd. (Ueda, Nagano, Japan), was used for all experiments. Silkworm larvae were reared on the artificial diet Silkmate 2 S (Nosan, Tsukuba, Ibaraki, Japan). Insects were maintained at 25 °C with a 12-h light/dark cycle.

### Chemicals

20E was purchased from Sigma-Aldrich (St. Louis, MO, USA) and dissolved in 10% isopropanol to make a 1-mg/ml stock solution. MnTBAP, a known SOD mimic, was purchased from Santa Cruz Biotechnologies, Inc, and dissolved in 0.1 M NaOH to make a 21.35-mg/ml stock solution. TMPyP was purchased from Sigma-Aldrich and dissolved in water to make a 62-mg/ml stock solution. DHE and Hoechst 33342 were purchased from Sigma-Aldrich and dissolved in DMSO or water, respectively, to make 10 mM stock solutions.

### 20E, MnTBAP, or TMPyP injection into *B. mori* larvae

The 1-mg/ml stock solution of 20E was adjusted to 5, 10, 20, 50, 100, or 200 µg/ml with 10% isopropanol, and 50 µL of each diluted solution was injected into the hemocoel of day 4 of fifth instar larvae. Thus, the final concentration injected was 0.25, 0.50, 1.0, 2.5, 5.0, or 10 µg/larva, respectively. The control larva was injected with only 50 µl of 10% isopropanol. After 24 or 48 h, the fat body was dissected from the larvae in each group.

The 21.35-mg/ml stock solution of MnTBAP was adjusted to 2.84, 5.68, or 11.4 mol/ml with 0.1 M NaOH. The 62-mg/ml stock solution of TMPyP was adjusted to 2.84, 5.68, 11.4, or 22.8 mol/ml with distilled water. On the sixth day, fifth instar larvae were anaesthetised with ice, and then 50 µL of each diluted solution of MnTBAP and TMPyP were injected intrahemocoelically using a 30 G needle (No.30 0.30 × 12 mm, Dentronics Co. Ltd., Tokyo, Japan). Thus, the final concentration injected was 0.142, 0.284, 0.568, or 1.14 mol/larva, respectively. The control larva was injected with only 50 µl of 0.1 M NaOH or distilled water. Pupation was observed after 5 days.

### Preparation of antibodies against BmSOD1 and BmSOD2

Recombinant BmSOD1 (rBmSOD1) and recombinant BmSOD2 (rBmSOD2) were prepared according to the methods described in our previous paper^11^, and they were purified using His GraviTrap (GE Healthcare Co. Ltd., Buckinghamshire, UK), according to manufacturer’s protocol. Antiserums for immunoblotting were raised for 6 weeks in female ICR mouse (Japan SLC, Inc., Shizuoka, Japan) by subcutaneous injection of 200 µg of rBmSOD1 or rBmSOD2 and TiterMax® Gold (TiterMax USA, Inc., Norcross, GA, USA) mixture. Booster immunization was performed twice 2 weeks after the prime immunization. These antiserums were collected and stored at −80 °C until use.

### Immunoblotting

Fat body samples were collected from four individual larvae (n = 4). To prepare protein extracts, the fat bodies were homogenized with a lysis buffer composed of 10 mM Tris-HCl, pH 7.5, and 130 mM NaCl, and supplemented with a protease inhibitor cocktail (Sigma-Aldrich). The protein extracts were centrifuged at 15,000 × g for 30 min at 4 °C. The protein concentration was determined using a BCA protein assay kit (Thermo Fisher Scientific, Inc., Waltham, MA, USA).

To identify the presence of BmSOD1 and BmSOD2 proteins, protein samples (5 or 10 μg) were separated on SDS-PAGE and transferred to nitrocellulose membranes (Bio-Rad Laboratories, Inc., Hercules, CA, USA) using the method used by *Towbin et al*.^40^. The membranes were incubated in blocking buffer composed of 5% milk and PBS, including 0.1% Tween 20 (PBS-T) for 1 h at room temperature (RT), then incubated in anti-BmSOD1 antiserum 1:10000, anti-BmSOD2 antiserum 1:10000, or anti-Actin antibody (ab1801; Abcam, Cambridge, UK) 1:1000 in blocking buffer overnight, and subsequently, washed with PBS-T for 10 min three times. The washed membranes were incubated with goat anti-rabbit IgG-conjugated horseradish peroxidase (HRP) 1:2000 (sc-2004; Santa Cruz Biotechnology, Inc), goat anti-mouse IgG-conjugated HRP 1:2000 (sc-2005; Santa Cruz Biotechnology, Inc) in blocking buffer for 1 h at RT and then washed with PBS-T for 10 min three times. The membranes were developed using a chemiluminescent substrate (Bio-Rad Laboratories, Inc) detected with Amersham Hyperfilm ECL (GE Healthcare). Immunoblotting was performed in triplicate as biological replications.

Antibodies were stripped by incubating the membranes at 50 °C for 30 min in stripping buffer composed of 62.5 mM Tris-HCl pH 6.8, 2% SDS, and 100 mM 2-mercaptoethanol and subsequently, these membranes were processed for relabeling with different antibodies. The band intensity was analyzed using ImageJ v. 1.51 s through Fiji ver. 1.0 (http://fiji.sc/). The expression level of both BmSOD proteins displayed relative expression levels. The expression level of both BmSOD proteins normalized with the expression of BmActin protein levels. Notably, BmActin was used as an endogenous control. Specificity for an anti-Actin antibody showed in the Supplementary Information.

### Measurement of SOD and AP activity

The SOD activity was measured using a SOD assay kit (Dojindo, Kumamoto, Japan) per the manufacturer’s instructions. In addition, the BmSOD2 activity was measured by blocking the Cu/Zn SOD and EC-SOD activity using diethyldithiocarbamate (DDC)^41,42^. To decide the appropriate concentration of DDC for measurement of BmSOD2 activity, we checked SOD activity with several doses of DDC (Supplementary Fig. S8). The total SOD activity fraction includes other Cu/Zn SOD and EC-SOD activity because *B. mori* has six types of Cu/Zn SODs^10^. However, we assessed the expression level of each SOD in the fat body by RNA-Seq and established that the expression level of other Cu/Zn SODs was very low^10^. Furthermore, the expression of *C. elegans* SOD1 mRNA covers in approximately 80% of the expression of all *C. elegans* SODs mRNA, and it is considered that *C. elegans* SOD1 activity contributes 80% of total SOD activity^43^. Hence, we considered that the majority of the total SOD activity was derived from SOD1 in the fat body, which was regarded as SOD1 activity. To measure the SOD2 activity, we added 1-mM DDC to the protein extract and then incubated it at 37 °C for 20 min. The value of the BmSOD1 activity was evaluated by subtracting the value of the BmSOD2 activity from the total SOD activity. Acid phosphatase activity was measured using an Acid Phosphatase assay kit (Abcam). These assays were performed according to the manufacturer’s instructions on three biological replicates. Total proteins (1 mg) were applied for each assay.

### DHE staining for evaluation of the amount of superoxide in the fat body

To evaluate the amount of superoxide in the fat body, we performed DHE staining as a modification of the protocol described by Owusu-Ansah *et al*.^44^. The fat body was dissected from four individual *B. mori* and mixed (n = 4). The dissected fat body was minced with scissors 8–10 times/min for 30 s in sterilized PBS, and the minced fat body was transferred to new PBS. The minced fat body was stained with 20 µM Hoechst 33342 (Sigma-Aldrich) diluted in PBS for 10 min at 25 °C using a rotator, by shading, and then stained with 30 µM dihydroethidium (DHE, Sigma-Aldrich) diluted with Grace’s insect medium (Thermo Fisher Scientific, Inc.) for 5 min at 25 °C using rotator under shading condition. The stained fat body was washed with Grace’s insect medium three times for five minutes. DHE-stained fat bodies were observed with an LSM 710 confocal scanning microscope (Carl Zeiss, Oberkochen, Germany). The excitation and emission wavelength were λex; 535, λem; 610 nm. Fluorescence intensities were quantified using ImageJ v. 1.51 s through Fiji ver. 1.0 (http://fiji.sc/). All observations were performed under the same conditions in triplicate as biological replications.

### RNA purification and quantitative RT-PCR

Fat body samples were weighed and homogenized with lysis buffer from a PureLink® RNA extraction kit (Thermo Fisher Scientific, Inc.) and then centrifuged at 13,000 × g for 10 min. Next, the supernatants were collected and processed for RNA purification, according to the manufacturer’s instructions. Purified total RNA (1 μg) was processed for quantitative RT-PCR (qRT-PCR).

One-step RT-PCR was performed using 20 μl of reaction volumes with 1 μg of RNA template and custom-made primers and probes (Table S1) from the TaqMan RNA-to-CT 1-Step Kit (Thermo Fisher Scientific, Inc.), in accordance with manufacturer instructions. qRT-PCR was performed on a Step One Plus Real-Time PCR System (Thermo Fisher Scientific, Inc.) following the Delta-Delta Ct method. 18 s ribosomal RNA (GeneID: 84310305) was used as an endogenous reference for the standardization of expression levels of RNA, and all data were calibrated against universal reference data. Relative quantification (RQ) values represent the relative expression level against a reference sample. All samples were assayed in triplicate as technical replications.

### Analysis of microarray data

GSE55816 and GSE23424 were obtained from the public database NCBI GEO (http://www.ncbi.nlm.nih.gov/geo/). To functionally annotate silkworm genes, silkworm genes homologous to fly genes were identified by conducting a systematic BLAST search (tblastx) with a cut-off E-value of significant homology at 1e-10 for the conversion table [query: silkworm transcript from KAIKObase (http://sgp.dna.affrc.go.jp/); database: whole-fly transcript from the Ensembl database (http://ensembl.org/)]. We used probe IDs of each platform for the assignment table. Probe IDs of each platform were converted into KAIKObase transcript IDs by conducting a systematic BLAST search (blastn). Two-fold upregulated gene lists were extracted from each data set and finally converted into fly transcript IDs from the assignment and conversion table. The converted two-fold upregulated gene IDs list was applied to PANTHER (http://www.pantherdb.org/) and DAVID (http://david.ncifcrf.gov/) for gene enrichment analysis (cut-off threshold: count ≥2 and *P* < 0.05).

### Statistical analysis

Statistical significance was determined by two-tailed Student’s *t*-test using Excel (Microsoft, Redmond, WA, USA). *P* values of < 0.05 were considered to be significant. Statistical analyses of pathways were automatically calculated by a default method in each pathway database.

Statistical analyses of the dose-response of SOD mimic injection were calculated by JMP 10.0 software (SAS Japan co. ltd., Tokyo, Japan) using fit to the general linear model in a default parameter.

### Ethics approval and consent to participate

The study protocol for the experimental use of the animals was approved by the ethics committee of Tokyo University of Agriculture and Technology (Approval ID, 26–40).

## Supplementary information

Supplementary material

## References

[1] Korsloot, A., Gestel, C. A. M. & Straalen, N. M. Environmental stress and cellular response in arthropods. 1–58 (CRC press 2004).

[2] Yu BP (1994). Cellular defenses against damage from reactive oxygen species. Physiol Rev..

[3] Fridovich I (1975). Superoxide dismutases. Annu Rev Biochem..

[4] Zelko IN, Mariani TJ, Folz RJ (2002). Superoxide dismutase multigene family: a comparison of the CuZn-SOD (SOD1), Mn-SOD (SOD2), and EC-SOD (SOD3) gene structures, evolution, and expression. Free Radic Biol Med..

[5] Mita K (2004). The genome sequence of silkworm, *Bombyx mori*. DNA Res..

[6] Tabunoki H (2013). Identification of key uric acid synthesis pathway in a unique mutant silkworm *Bombyx mori* model of Parkinson’s disease. PLoS One.

[7] Yamamoto K (2005). Superoxide dismutase from the silkworm, *Bombyx mori*: sequence, distribution, and overexpression. Biosci Biotechnol Biochem..

[8] Yamamoto K (2005). Molecular and biochemical characterization of manganese-containing superoxide dismutase from the silkworm, *Bombyx mori*. Comp Biochem Physiol B Biochem Mol Biol..

[9] Isobe M (2006). The molecular mechanism of the termination of insect diapause, part 1: A timer protein, TIME-EA4, in the diapause eggs of the silkworm *Bombyx mori* is a metallo-glycoprotein. Chembiochem.

[10] Kobayashi Y (2019). Comparative analysis of seven types of superoxide dismutases for their ability to respond to oxidative stress in Bombyx mori. Sci Rep..

[11] Nojima Y (2015). Superoxide dismutases, SOD1 and SOD2, play a distinct role in the fat body during pupation in silkworm *Bombyx mori*. PLoS One.

[12] Callier V, Nijhout HF (2011). Control of body size by oxygen supply reveals size-dependent and size-independent mechanisms of molting and metamorphosis. Proc Natl Acad Sci USA.

[13] Inoue M (2004). Free radical theory of apoptosis and metamorphosis. Redox Rep..

[14] Tian L (2013). 20-Hydroxyecdysone upregulates Atg genes to induce autophagy in the *Bombyx* fat body. Autophagy.

[15] Huang N, Clem RJ, Rohrmann GF (2011). Characterization of cDNAs encoding p53 of *Bombyx mori* and Spodoptera frugiperda. Insect Biochem Mol Biol..

[16] Liu M (2014). Cloning and expression characteristics of the notch-associated gene BmE(spl)mγ from silkworm, *Bombyx mori*. Appl Biochem Biotechnol..

[17] Li K (2015). Bombyx E75 isoforms display stage- and tissue-specific responses to 20-hydroxyecdysone. Sci Rep..

[18] Nishita Y (2014). Ecdysone response elements in the distal promoter of the Bombyx Broad-Complex gene, BmBR-C. Insect Mol Biol..

[19] Joe Y (2012). Salvianolic acid B exerts vasoprotective effects through the modulation of heme oxygenase-1 and arginase activities. J Pharmacol Exp Ther..

[20] Al-Kafaji G, Golbahar J (2013). High glucose-induced oxidative stress increases the copy number of mitochondrial DNA in human mesangial cells. Biomed Res Int..

[21] Pizova K (2015). C-MYC and C-FOS expression changes and cellular aspects of the photodynamic reaction with photosensitizers TMPyP and ClAlPcS2. J Photochem Photobiol B.

[22] Liu W (2012). Functional analyses in the silkworm, *Bombyx mori*, support a role for Notch signaling in appendage development but not the groucho-dependent pair-rule process. J Exp Zool B Mol Dev Evol..

[23] Liu W (2013). Bmdelta phenotype implies involvement of Notch signaling in body segmentation and appendage development of silkworm, *Bombyx mori*. Arthropod Struct Dev..

[24] Koch U, Lehal R, Radtke F (2013). Stem cells living with a Notch. Development..

[25] Small C (2014). An unexpected link between notch signaling and ROS in restricting the differentiation of hematopoietic progenitors in Drosophila. Genetics..

[26] Cai W (2016). Notch1 pathway protects against burn-induced myocardial injury by repressing reactive oxygen species production through JAK2/STAT3 signaling. Oxid Med Cell Longev..

[27] Cai WX (2014). Inhibition of Notch signaling leads to increased intracellular ROS by up-regulating Nox4 expression in primary HUVECs. Cell Immunol..

[28] Huang J, Wu S, Barrera J, Matthews K, Pan D (2005). The Hippo signaling pathway coordinately regulates cell proliferation and apoptosis by inactivating Yorkie, the Drosophila Homolog of YAP. Cell.

[29] Kaneko Y, Yasanga T, Suzuki M, Sakurai S (2011). Larval fat body cells die during the early pupal stage in the frame of metamorphosis remodelation in *Bombyx mori*. J Insect Physiol..

[30] Tian L, Liu S, Liu H, Li S (2012). 20-hydroxyecdysone upregulates apoptotic genes and induces apoptosis in the Bombyx fat body. Arch Insect Biochem Physiol..

[31] Gibbens YY, Warren JT, Gilbert LI, O’Connor MB (2011). Neuroendocrine regulation of Drosophila metamorphosis requires TGFbeta/Activin signaling. Development..

[32] Yamanaka N, Rewitz KF, O’Connor MB (2013). Ecdysone control of developmental transitions: lessons from Drosophila research. Annu Rev Entomol.

[33] Kamimura M, Tomita S, Kiuchi M, Fujiwara H (1997). Tissue-specific and stage-specific expression of two silkworm ecdysone receptor isoforms–ecdysteroid-dependent transcription in cultured anterior silk glands. Eur J Biochem..

[34] Mizoguchi A, Dedos SG, Fugo H, Kataoka H (2002). Basic pattern of fluctuation in hemolymph PTTH titers during larval-pupal and pupal-adult development of the silkworm, *Bombyx mori*. Gen Comp Endocrinol..

[35] Zeng B (2017). The FOXO transcription factor controls insect growth and development by regulating juvenile hormone degradation in the silkworm, Bombyx mori. J Biol Chem..

[36] Li L, Chen Y, Gibson SB (2013). Starvation-induced autophagy is regulated by mitochondrial reactive oxygen species leading to AMPK activation. Cell Signal..

[37] Tabunoki H, Gorman MJ, Dittmer NT, Kanost MR (2016). Superoxide dismutase 2 knockdown leads to defects in locomotor activity, sensitivity to paraquat, and increased cuticle pigmentation in *Tribolium castaneum*. Sci Rep..

[38] Jia H (2014). Characterization of a mitochondrial manganese superoxide dismutase gene from Apis cerana cerana and its role in oxidative stress. J Insect Physiol..

[39] SAFETY DATA SHEET MnTBAP chloride (SC-221954) Santa Cruz Biotechnology, Inc. Revision date 13-Dec-2016 Version 1.3.

[40] Towbin H, Staehelin T, Gordon J (1979). Electrophoretic transfer of proteins from polyacrylamide gels to nitrocellulose sheets: procedure and some applications. Proc Natl Acad Sci USA.

[41] Ahmad, S. Oxidative stress and Antioxidant Defences in Biology, 238–244 (Springer 1995).

[42] Misra HP (1979). Reaction of copper-zinc superoxide dismutase with diethyldithiocarbamate. J Biol Chem..

[43] Doonan R (2008). Against the oxidative damage theory of aging: superoxide dismutases protect against oxidative stress but have little or no effect on life span in Caenorhabditis elegans. Genes Dev..

[44] Edward Owusu-Ansah, E., Yavari, A. & Banerjee, U. A protocol for *in vivo* detection of reactive oxygen species. *Protocol Exchange* **414** (2008).

