# Supplementary material for: Superoxide dismutase down-regulation and the oxidative stress is required to initiate pupation in Bombyx mori
Source: Sci Rep. 2019 Oct 11;9:14693. doi: 10.1038/s41598-019-51163-3 (PMC6788986; doi:10.1038/s41598-019-51163-3)

Superoxide dismutase down-regulation and the oxidative stress is required to initiate pupation in *Bombyx mori*

Yosui Nojima[1], Hidemasa Bono[2], Takeshi Yokoyama[1,3], Kikuo Iwabuchi[3], Ryoichi Sato[4], Katsuhiko Arai[5], and Hiroko Tabunoki[1,3*]

[1]Department of United Graduate School of Agricultural Science, Tokyo University of Agriculture and Technology, 3-5-8 Saiwai-cho, Fuchu, Tokyo, 183-8509, Japan

[2]Database Center for Life Science (DBCLS), Joint Support-Center for Data Science Research, Research Organization of Information and Systems (ROIS), Yata 1111, Mishima, Shizuoka 411-8540, Japan

[3]Department of Science of Biological Production, Graduate School of Agriculture, Tokyo University of Agriculture and Technology, 3-5-8 Saiwai-cho, Fuchu, Tokyo, 183-8509, Japan

[4]Bio-Applications and Systems Engineering, Tokyo University of Agriculture and Technology, Koganei, Tokyo 184-8588, Japan

[5]Department of Tissue Physiology, Tokyo University of Agriculture and Technology, Fuchu, Tokyo 183-8509, Japan

Yosui Nojima: yosui.nojima@gmail.com

Hidemasa Bono: bono@dbcls.rois.ac.jp

Takeshi Yokoyama: ty.kaiko@cc.tuat.ac.jp

Kikuo Iwabuchi:kikkuo@cc.tuat.ac.jp

Ryoichi Sato:ryoichi@cc.tuat.ac.jp

Katsuhiko Arai: karai@cc.tuat.ac.jp

Hiroko Tabunoki: h_tabuno@cc.tuat.ac.jp

*To whom correspondence should be addressed: Tel: +81-42-367-5613; Fax: +81-42-367-5613; E-mail: h_tabuno@cc.tuat.ac.jp

Supplementary information

Methods

Cell culture

BmN cell derived from silkworm ovarian tissue was obtained from RIKEN BRC (RCB0457) and maintained at 25°C with 10% fetal bovine serum (FBS) containing TC-100 medium (Appli Chem Co., Ltd., Darmstadt, Germany). The HepG2 cell was obtained from RIKEN BRC (RCB1886) and maintained at 37°C under 5% CO_2 in Dulbecco's Modified Eagle's medium (DMEM; Thermo Fisher Scientific Inc.) supplemented with 10% fetal bovine serum (FBS), 100 U/ml penicillin, and 100 µg/ml streptomycin (Thermo Fisher Scientific, Inc.).

Isolation of the cytosol and mitochondrial fraction from the fat body tissue

The cytosol and mitochondrial fractions were isolated from the fat body samples dissected from day 4 fifth instar larvae using the method used by Clayton *et al.* [1]. The isolated mitochondrial pellet was re-suspended in a lysis buffer comprising 2% CHAPS and TBE. The re-suspended mitochondria pellet was centrifuged at 15,000 g for 10 min at 4°C. The supernatant was collected and stored at −80°C until used for immunoblotting.

Immunoblotting

To prepare protein extracts, tissues and cells were homogenized with a lysis buffer composed of 10 mM Tris-HCl (pH 7.5) and 130 mM NaCl and supplemented with a protease inhibitor cocktail (Sigma-Aldrich). The protein extracts were centrifuged at 15,000 × g for 30 min at 4°C. The protein concentration was determined using a BCA protein assay kit (Thermo Fisher Scientific Inc., Waltham, MA, USA).

To identify the presence of BmSOD1 and BmSOD2 proteins in the tissues and cells, protein samples (5 or 10 μg) were separated on SDS-PAGE and transferred to nitrocellulose membranes (Bio-Rad Laboratories, Inc., Hercules, CA, USA) using the method used by Towbin *et al.* [2]. The membranes were incubated with blocking buffer composed of 5% milk and PBS, including 0.1% Tween 20 (PBS-T) for 1 h at room temperature. Subsequently, the membranes were incubated with anti-BmSOD1 serum 1:10000, anti-BmSOD2 serum 1:10000, anti-Actin antibody (ab1801; Abcam, Cambridge, UK) 1:1000, anti-Hsp60 antibody (sc-1052; Santa Cruz Biotechnology, Inc., Dallas, TX, USA) 1:5000, or anti-Xpress-tag antibody (R910-25; Thermo Fisher Scientific Inc.) 1:5000 in blocking buffer overnight and then washed with PBS-T for 10 min three times. After washing, the membranes were incubated with goat anti-rabbit IgG-conjugated horseradish peroxidase (HRP) 1:2000 (sc-2004; Santa Cruz Biotechnology, Inc), goat anti-mouse IgG-conjugated HRP 1:2000 (sc-2005; Santa Cruz

Biotechnology, Inc), or donkey anti-goat IgG-conjugated HRP 1:2000 (sc-2020, Santa Cruz Biotechnology, Inc) in blocking buffer for 1 h. The membranes were then washed with PBS-T for 10 min three times. Membranes were developed using a chemiluminescent substrate (Bio-Rad Laboratories, Inc), then chemiluminescence was detected using Amersham Hyperfilm ECL (GE Healthcare).

RT-PCR

The stage distribution of the BmAtg1 genes was determined using the fat body. The distribution of the BmAtg1 genes in the fat body from fifth instar larvae to the adult was determined. All samples were processed for extraction of total RNA and cDNA synthesis as previously described [3]. Reverse transcriptase (RT)-PCR was performed with specific primers (BmAtg1; 5’-CCCCGCCTATGTCTATGTTG-3’, 5’- ATCTGATGGGTGGGAGTACG-3’) using AmpliTaq Gold® 360 Master Mix (Thermo Fisher Scientific Inc.) according to the manufacturer’s protocol. *B. mori* 18s rRNA was used as an endogenous control.

Measurement of SOD activity

The SOD activity was measured using a SOD assay kit (Dojindo, Kumamoto, Japan) per the manufacturer’s instructions. In addition, the BmSOD2 activity was measured by blocking the Cu/Zn SOD and EC-SOD activity using diethyldithiocarbamate (DDC). To decide the appropriate

concentration of DDC using measurement BmSOD2 activity, we added with several doses of DDC (0, 0.5, 1.0, 2.0 mM) to the protein extract and then incubated it at 37˚C for 20 min, and then total SOD activity was measured.

Fig. S1 Specificity of the BmSOD1 and BmSOD2 antibody

(**a**) Specificity for anti-BmSOD1 antiserum and anti-BmSOD2 antiserum. Five microgram of fat body lysate (lane 1), 5 μg of BmN cell lysate (lane 2), 5 μg of HepG2 cell lysate (lane 3), 30 ng of purified recombinant BmSOD1 (lane 4), and 30 ng of purified recombinant BmSOD2 (lane 5) were separated on a 15% SDS-PAGE gel, transferred onto a nitrocellulose membrane, and probed with each antibody. These samples were reacted with anti-BmSOD1 antiserum (upper panel), anti-BmSOD2 antiserum (middle panel), and anti-Xpress antibody (lower panel). (**b**) Specificity for an anti-Actin antibody. Five micrograms of fat body lysate (lane 1), BmN cell lysate (lane 2), HepG2 cell lysate (lane 3), and *D. melanogaster* whole-body lysate (lane 4) were separated on a 15% SDS-PAGE gel, transferred onto a nitrocellulose membrane, and probed with the antibody. The black arrow indicates the molecular size of BmActin.

Fig. S2 Distribution of BmSOD1 and BmSOD2 in the fat body cell

The fraction was isolated from the fat body of day 4 fifth instar larva using the method used by Clayton et al. [1]. Five micrograms of the whole tissue lysate of fat body, cytosol fraction, and mitochondria fraction were separated on a 15% SDS-PAGE gel, transferred onto a nitrocellulose membrane, and probed with each antiserum or antibody. BmActin and BmHsp60 proteins were used as cytosol or mitochondria markers. W: whole tissue lysate, C: cytosol fraction, and M: mitochondria fraction

Fig. S3 Tissue distribution of BmSOD1 and BmSOD2 proteins from fifth instar larva to adult

Malpighian tubule (Mt), midgut (Mg), testis (Tes), and ovary (Ov) were used. Aliquots (10 μg) of each tissue lysate were separated by 15% SDS-PAGE; transferred to nitrocellulose; and probed with BmSOD1, BmSOD2, and BmActin antibodies. BmActin was used as an endogenous control.

Fig. S4 Tissue distribution of BmSOD1 and BmSOD2 proteins in the fourth instar larval developmental stage

Fat body (a), Malpighian tubule (b), midgut (c), were used. Aliquots (10 μg) of each tissue lysate were separated by 15% SDS-PAGE; transferred to nitrocellulose; and probed with BmSOD1, BmSOD2, and BmActin antibodies. BmActin was used as an endogenous control. 0 hour of day 1 fourth instar (day

1E), 0 hour of day 2 fourth instar (day 2E), 0 hour of day 3 fourth instar (day 3E), 12 hour of day 3 fourth instar (day 3L), 12 hour of day 4 fourth instar (day 4E), 12 hour of day 4 fourth instar (day 4L).

Fig. S5 Average of the body weight in Kinshu x Showa of the *B. mori*

Each of the body weight was measured and plotted on the graph. The day shows days after ecdysis in the fourth or fifth larval developmental stage. Error bars indicate SD (n = 6 to 14).

Fig. S6 Developmental stage expression patterns of BmAtg1 mRNAs as determined by RT-PCR

Fat bodies were dissected from *B. mori* larvae through larval to the adult developmental stage. mRNA expression of BmAtg1 examined by RT-PCR. 18s rRNA was used as the endogenous control.

Fig. S7 SOD mimic suppressed pupation in *B. mori* larvae

SOD mimic was injected into day 6 fifth instar larva at concentration of 0, 0.142, 0.284, or 0.568 mol/larva. The image shows the cocoon made by injected larvae as observed after 6 days (upper panel). The image shows injected larvae as observed after 7 days (lower panel).

Fig. S8 Examination of DDC concentration for evaluating BmSOD2 activity

SOD activities were measured in the protein extracts with the several doses of DDC, and the total SOD activities were plotted on the graph.

Table S1

Target	Probe (5’-3’)	Forward (5’-3’)	Reverse (5’-3’)
BmSOD1	TACGCCATGTCGGCGACCTCG	ATCATGGTGGTCCCAGTTCTG	CAGAGTCTTCAATTGCCTCAATGT
BmSOD2	CCACTCGATCTTTTGGCACAACCTGT	TCAATGGTGGTGGTCACATCA	AGGCTTGCCACCATTTGG
BmAtg1	AAACGAACCGGCTGCTCAGCGA	AGGCGACCTAGCGGATTATCT	TCTGCAAGCTGCGCTAAGAA
Bmp53	TCGAAATCCACAGCAAGGATACACACAAGA	CCCGGGCAATACAACTTCAG	TTCAGCTTGTGCGAGAGCAA
BmE(spl)mγ	CCTGCGCCTCAGACACATCAGGGT	ACTCCTCTGCAGCCCATAGC	ATCCTCTGGACGGGCATCA

BmE75A	CATCCCTTGTGACGTGCCGGC	TGGCAGTGTTGCGAGACAAG	TGAATATAGCACAGGAGTGCATGA
BmBr-C	ACCTCGCCGTGGTAGATGAACTCGAC	GGACAAGCTGTGCTGGTGAA	TCAAGACGTGGCGTACACAGA
18s rRNA	CCGCCCGTCGCTACTACCGATTG	CGTCCCTGCCCTTTGTACAC	CGGTCCGAAGACCTCACTAAAT

Probes and primer sets were custom designed with 5'-labeled 6-FAM and 3'-labeled TAMRA.

Chemical	Dose (mol/larva)	Number of larvae	Observation (count of pupation)	Observation after three days (count of the dead individual)
SOD mimic	0	3	3	0
	0.142	3	0	2
	0.284	3	0	3
	0.568	3	0	0
TMPyP	0	3	3	0
	0.142	3	3	0
	0.284	3	3	0
	0.568	3	0	0
	1.140	3	0	1

Table S2 Observation of pupation in treatment with SOD mimic *B. mori* larva

Reference

1. Clayton DA, Shadel GS: Isolation of mitochondria from animal tissue. Cold Spring Harb Protoc 2014, 2014(10):pdb.prot080010.
2. Towbin H, Staehelin T, Gordon J: Electrophoretic transfer of proteins from polyacrylamide gels to nitrocellulose sheets: procedure and some applications. Proc Natl Acad Sci U S A 1979, 76(9):4350-4354.
3. Nojima, Y. *et al.* Superoxide dismutases, SOD1 and SOD2, play a distinct role in the fat body during pupation in silkworm *Bombyx mori*. *PLoS One* **10**, e0116007 (2015)

Fig. S1

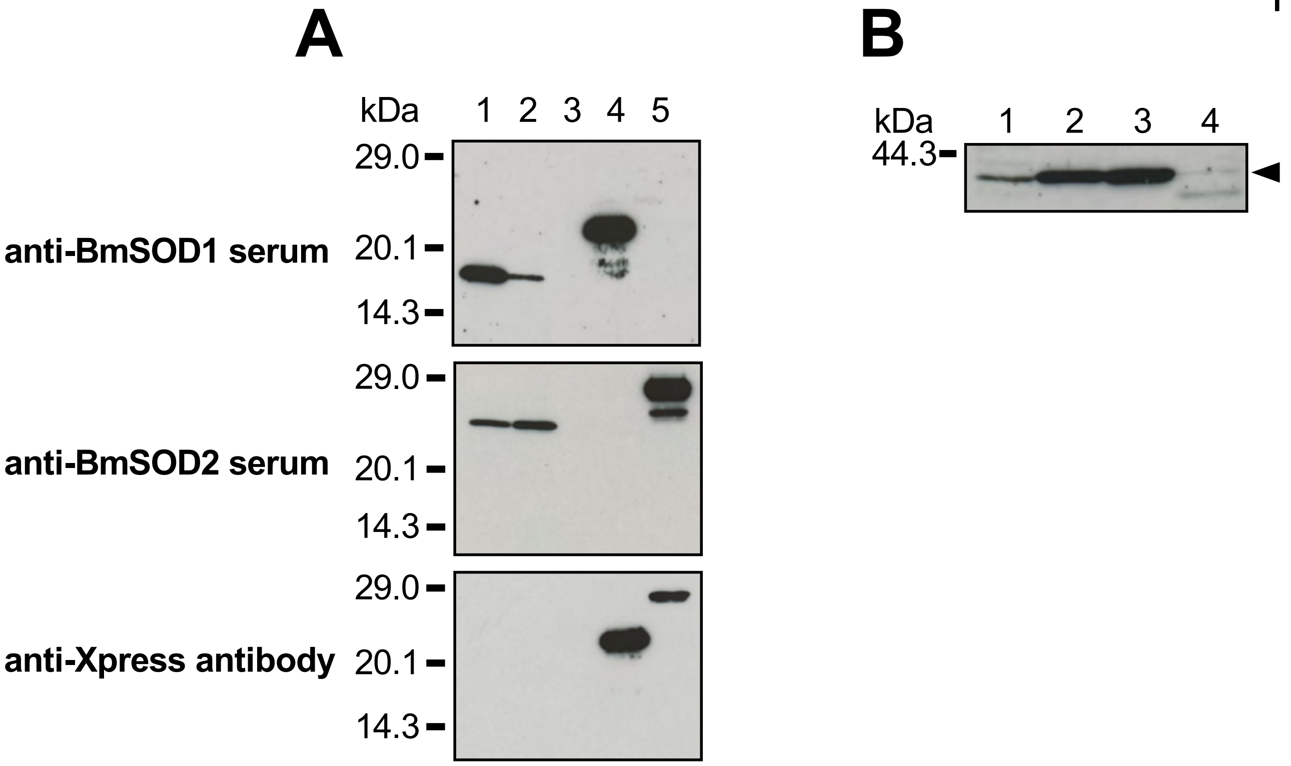

A
kDa
1 2 3 4 5
anti-BmSOD1 serum
29.0
20.1
14.3
anti-BmSOD2 serum
29.0
20.1
14.3
anti-Xpress antibody
29.0
20.1
14.3
B
kDa
1 2 3 4
44.3

Fig. S2

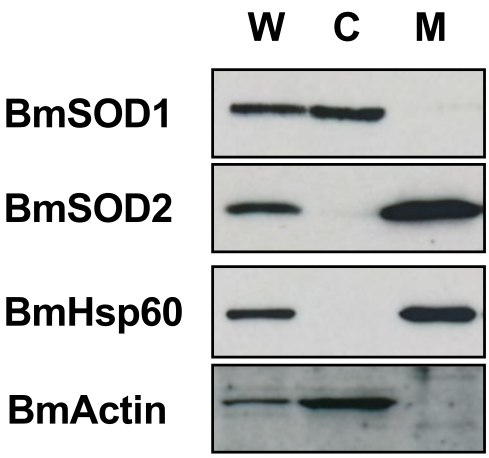

Fig. S3

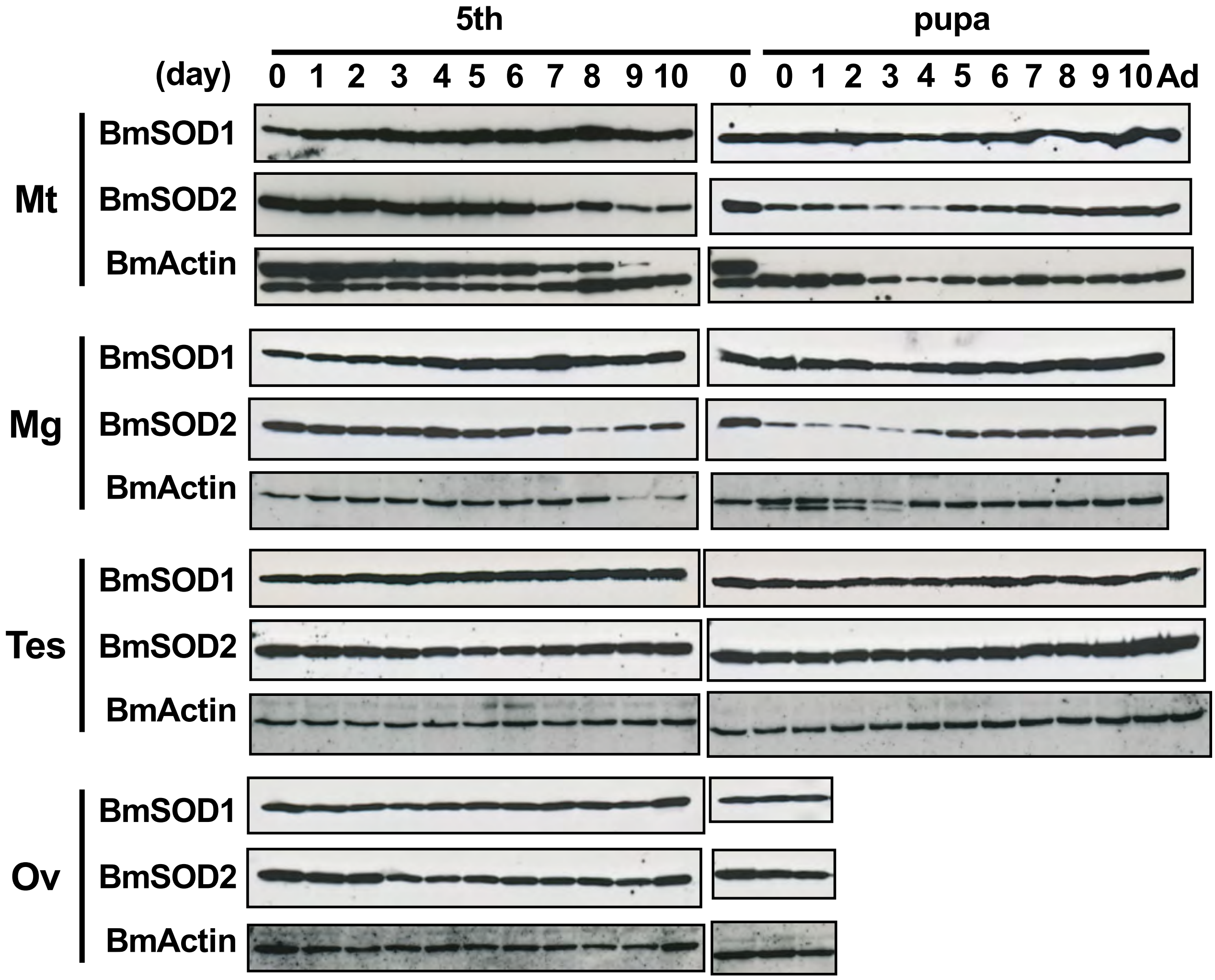

5th
pupa
(day) 0 1 2 3 4 5 6 7 8 9 10 0 0 1 2 3 4 5 6 7 8 9 10 Ad
Mt
BmSOD1
BmSOD2
BmActin
Mg
BmSOD1
BmSOD2
BmActin
Tes
BmSOD1
BmSOD2
BmActin
Ov
BmSOD1
BmSOD2
BmActin

Fig. S4

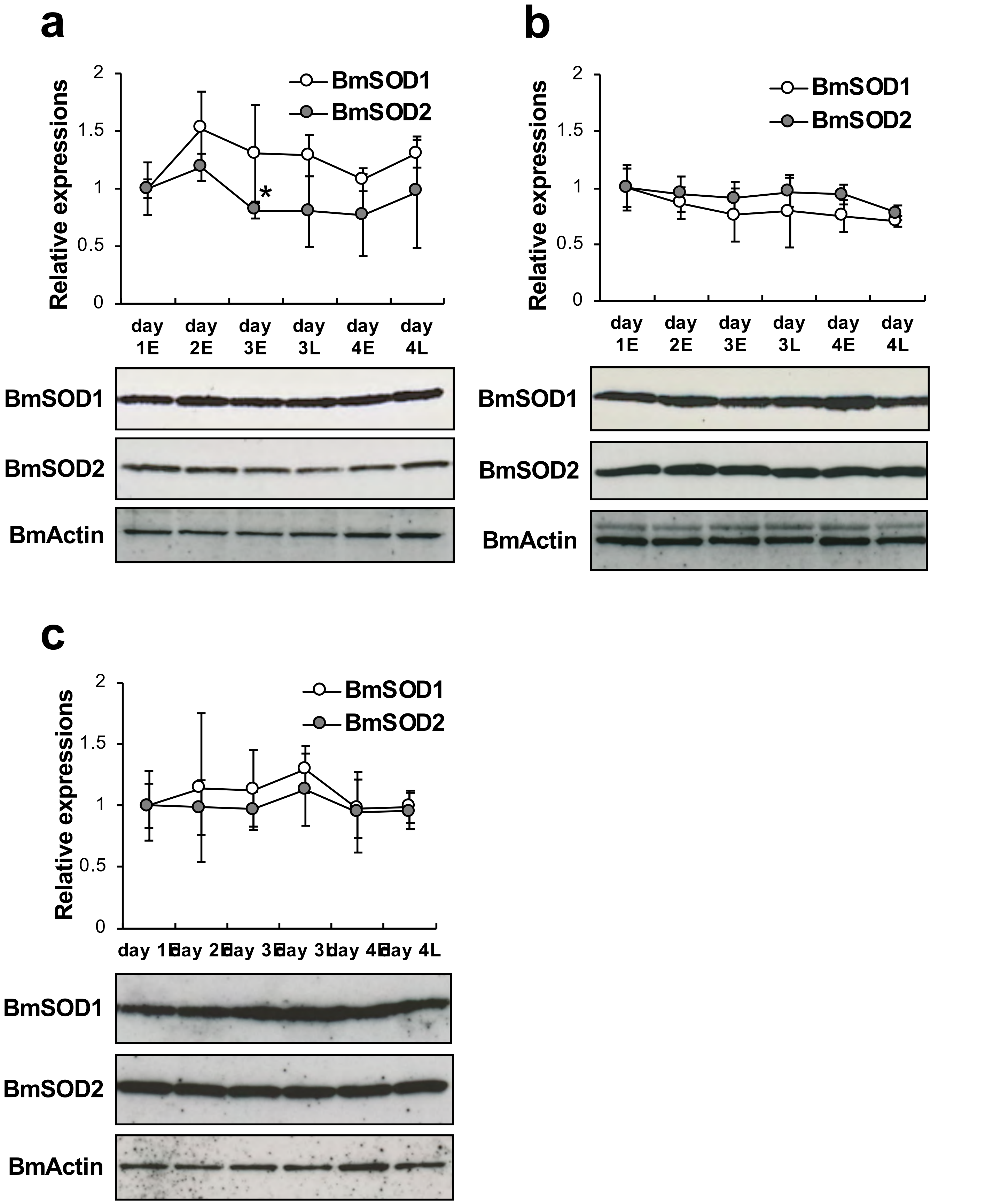

a
b
c
Relative expressions
BmSOD1
BmSOD2
BmActin
day 1E
day 2E
day 3E
day 3L
day 4E
day 4L
*

Fig. S5

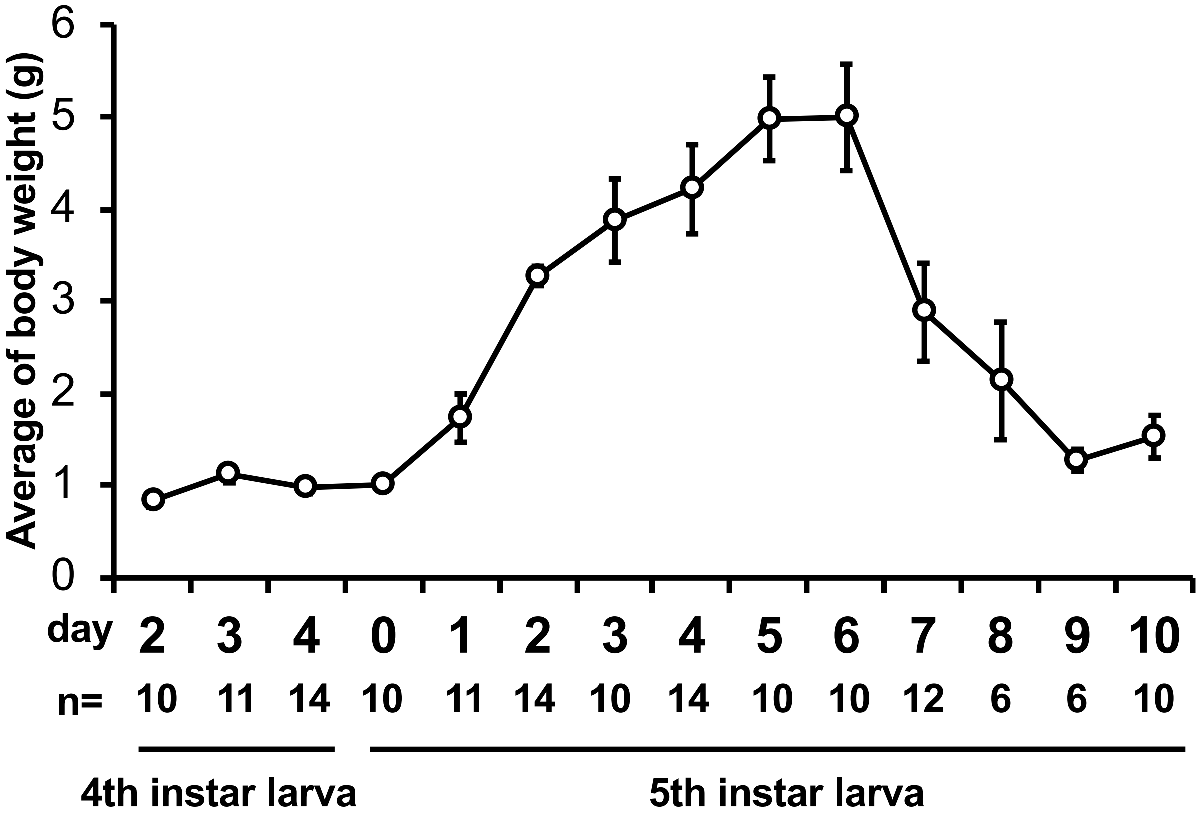
Average of body weight (g)
0
1
2
3
4
5
6
day
2 3 4 0 1 2 3 4 5 6 7 8 9 10
n=
10 11 14 10 11 14 10 14 10 10 12 6 6 10
4th instar larva
5th instar larva

Fig. S6

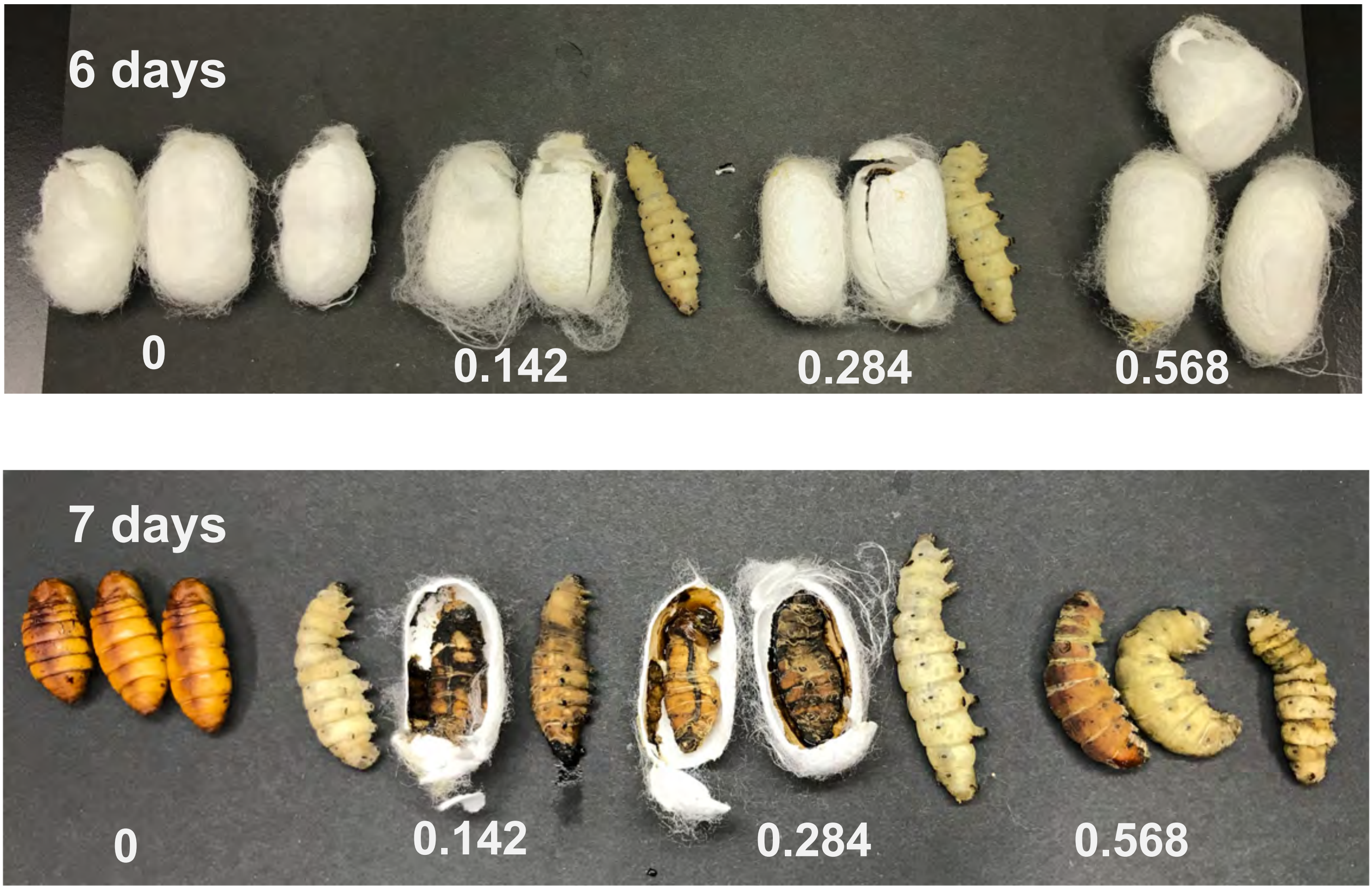

Fig. S7

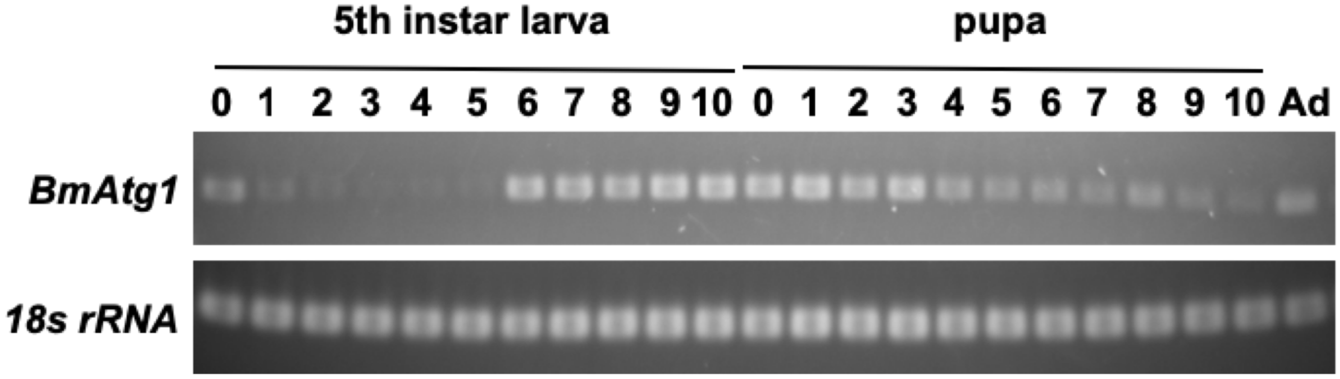
5th instar larva
pupa
0 1 2 3 4 5 6 7 8 9 10 0 1 2 3 4 5 6 7 8 9 10 Ad
BmAtg1
18s rRNA

Fig. S8

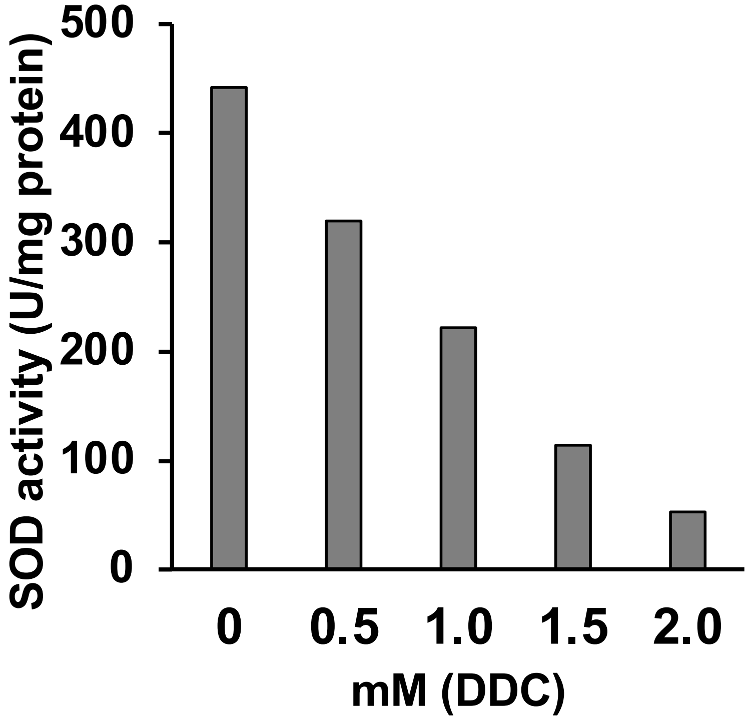

SOD activity (U/mg protein)
500
400
300
200
100
0
0
0.5
1.0
1.5
2.0
mM (DDC)

Figure2-A_5th-larva_BmActin

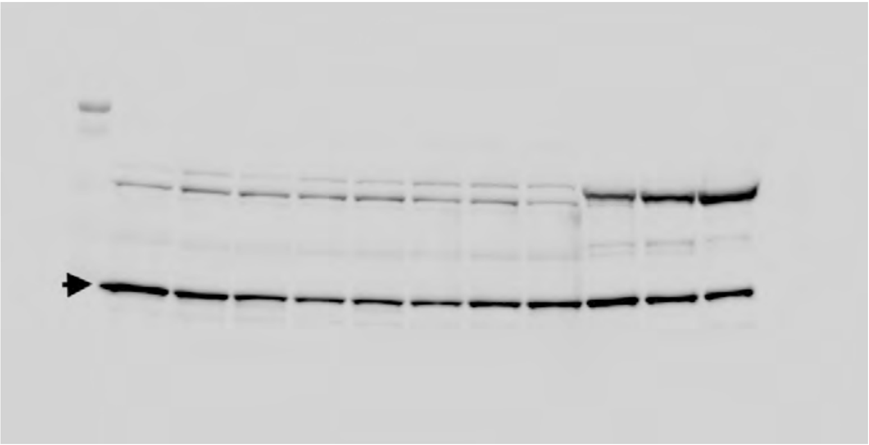

Figure2-A_5th-larva_BmSOD1

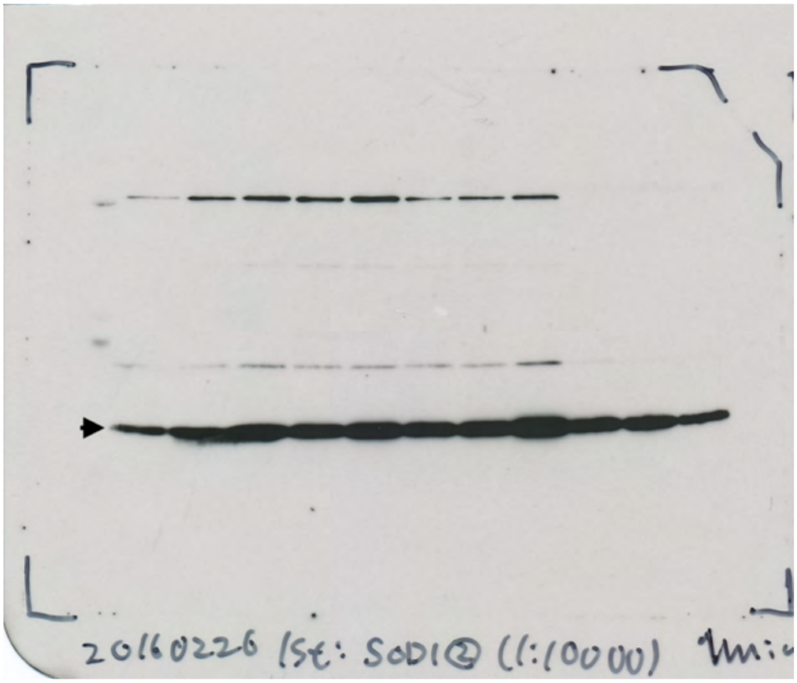

Figure2-A_5th-larva_BmSOD2

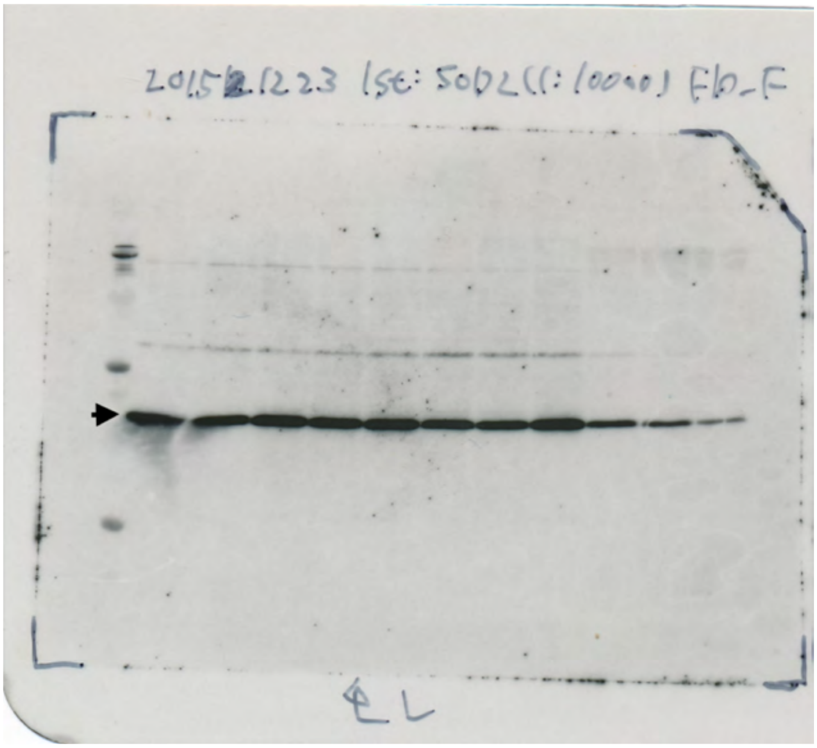

Figure2-A_pupa-adult_BmActin

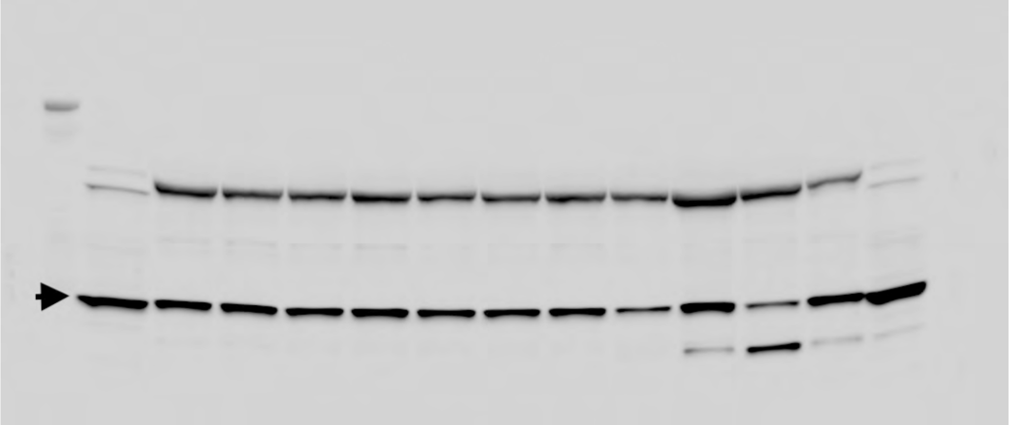

Figure2-A_pupa-adult_BmSOD1

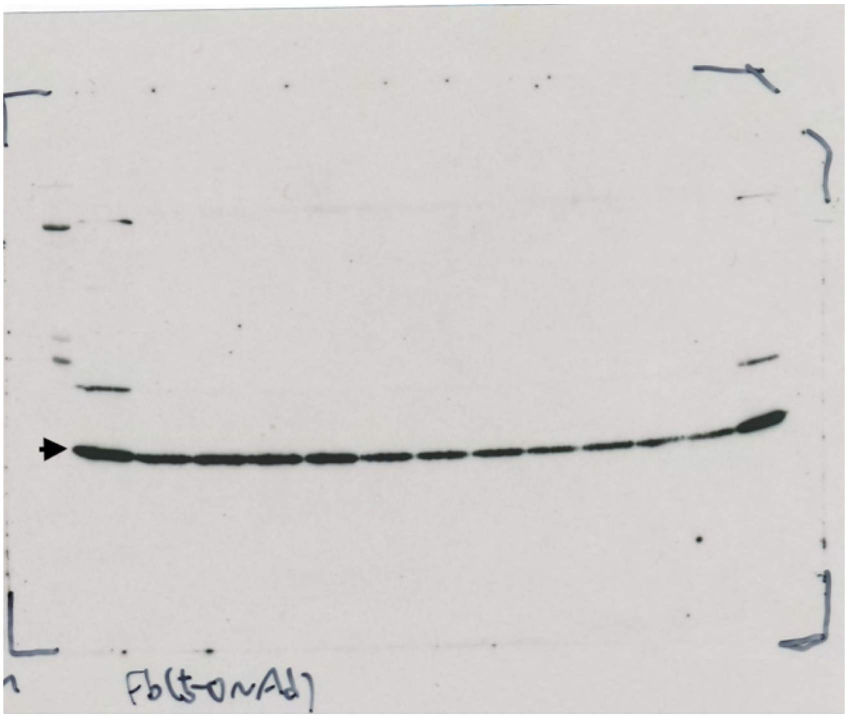

Figure2-A_pupa-adult_BmSOD2

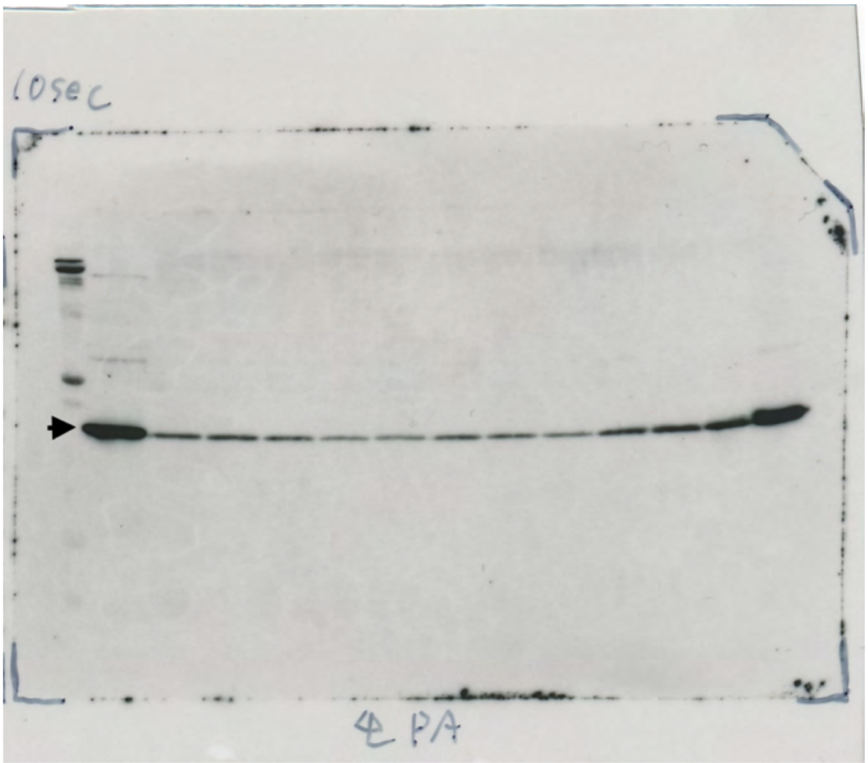

Figure4-C_BmActin

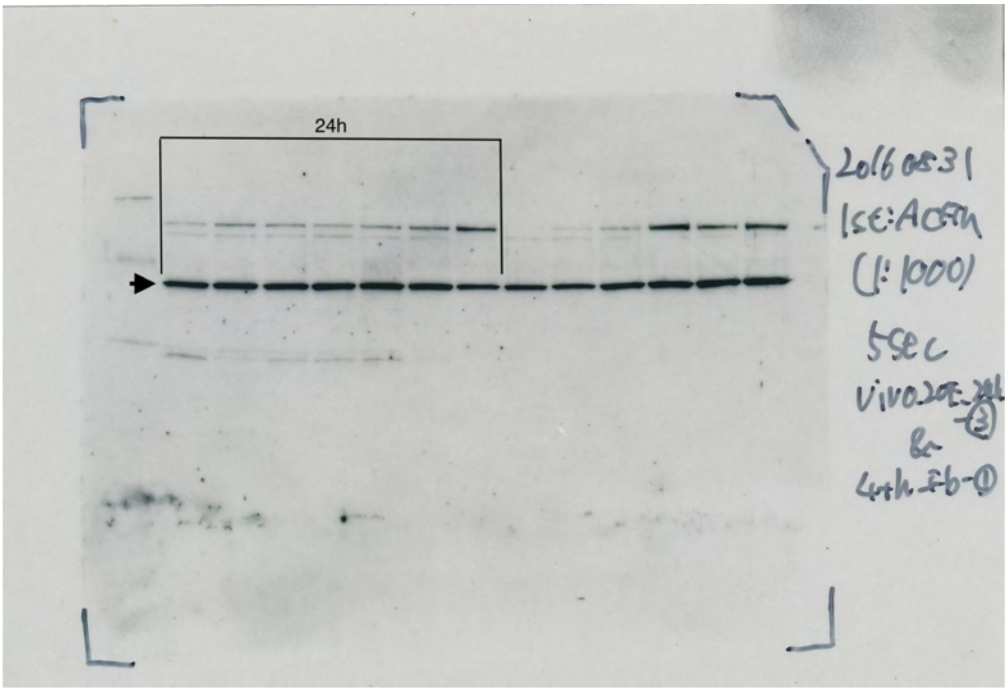

Figure4-C,D_BmSOD1

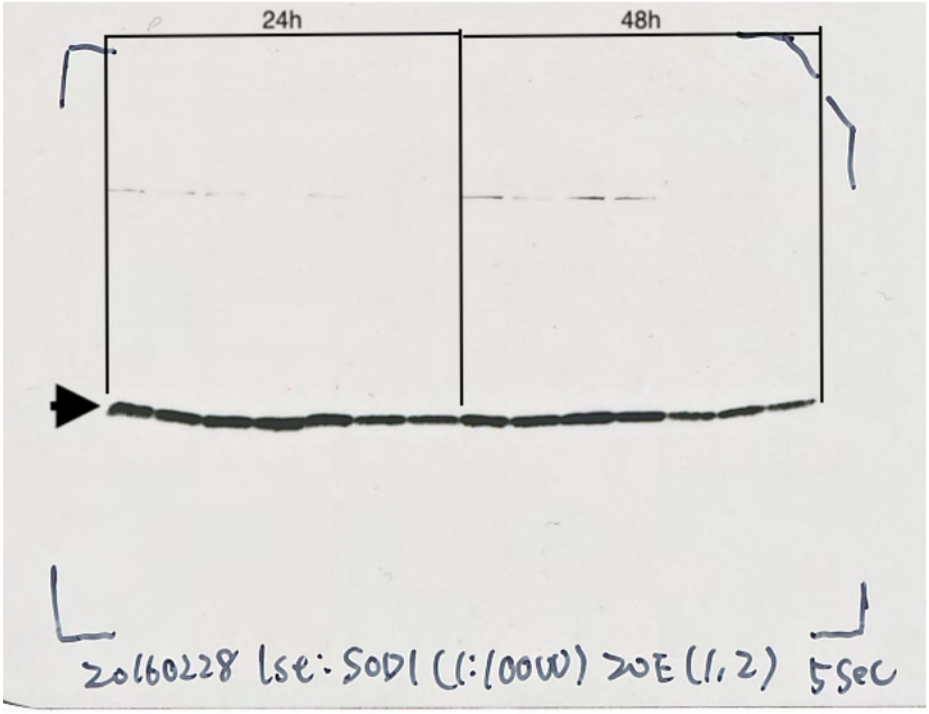

Figure4-C,D_BmSOD2

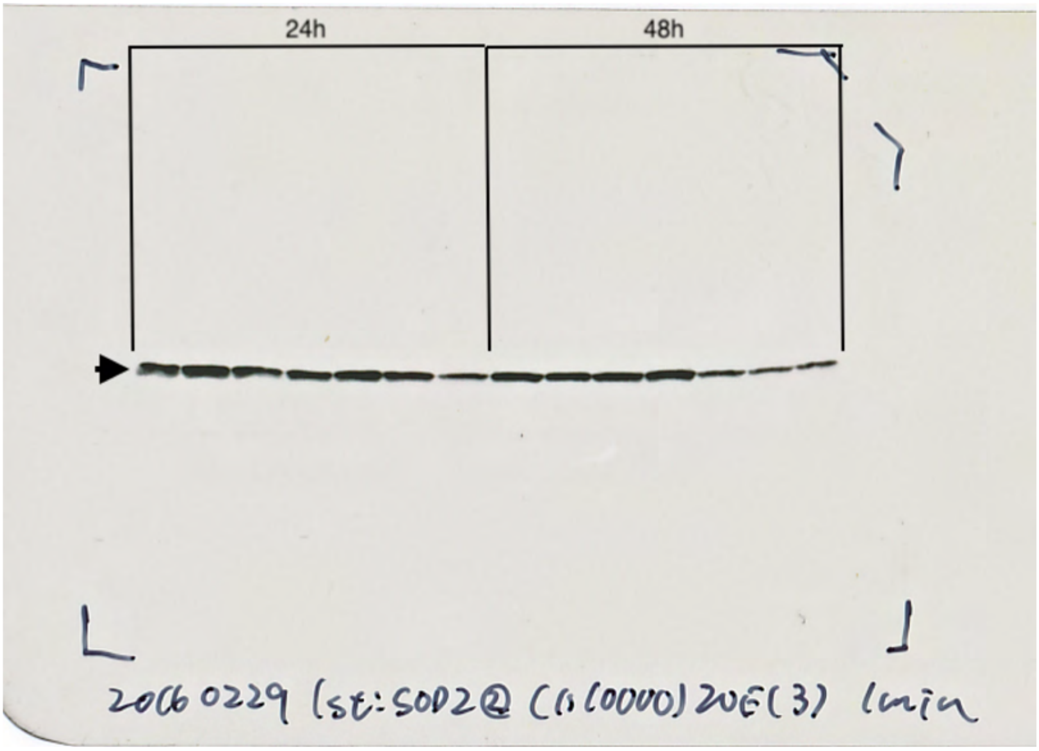

Figure4-D_BmActin

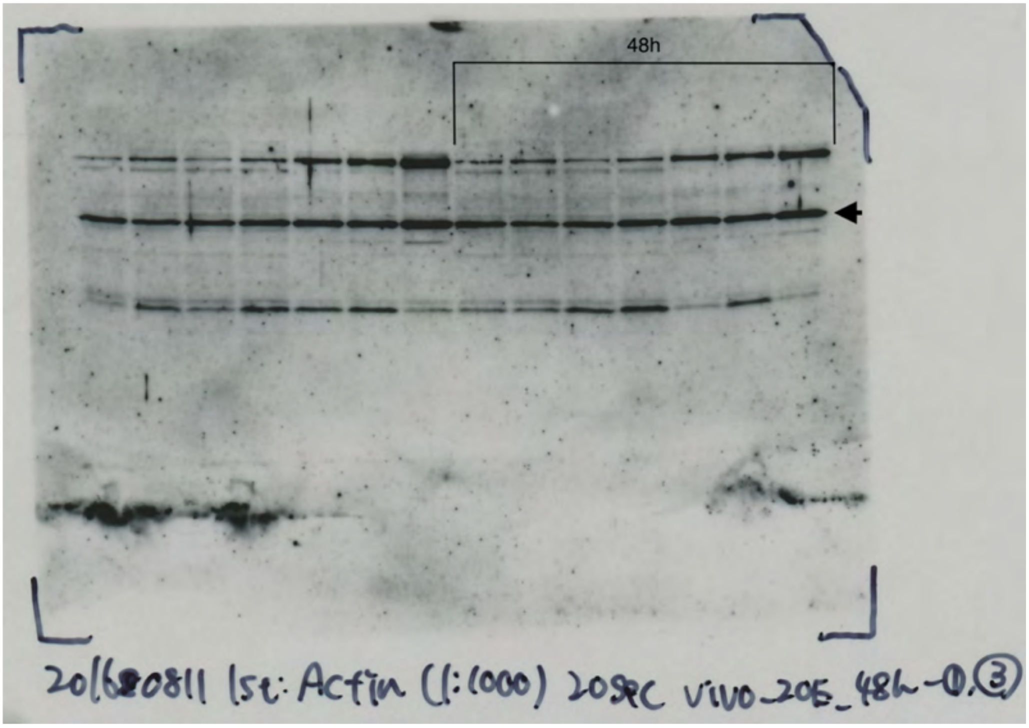

FigureS1-A_BmSOD1

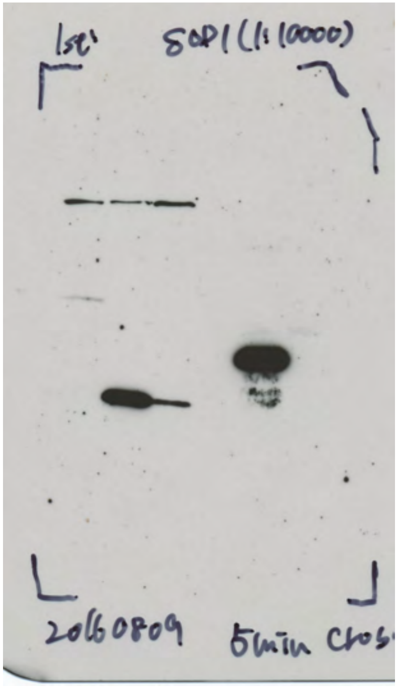

FigureS1-A_BmSOD2

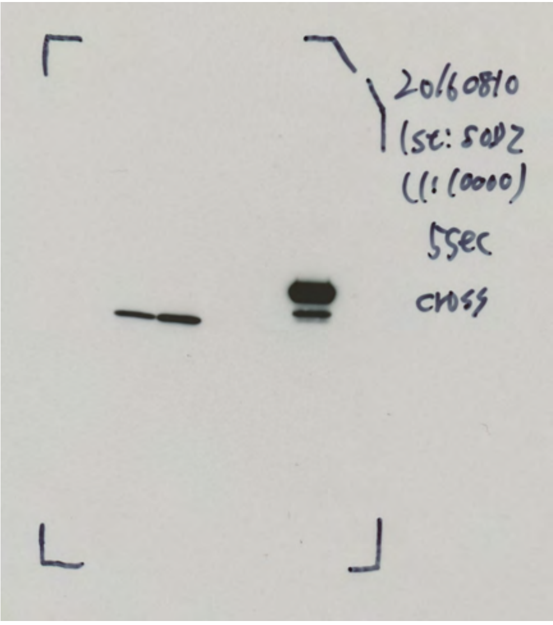

FigureS1-A_Xpress

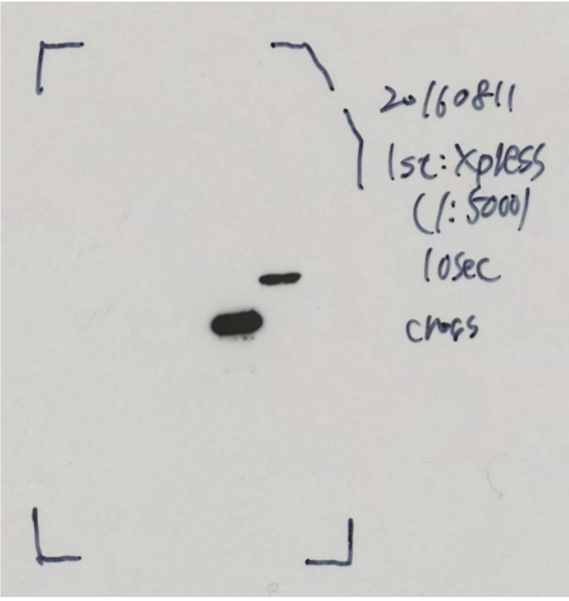

FigureS1-B

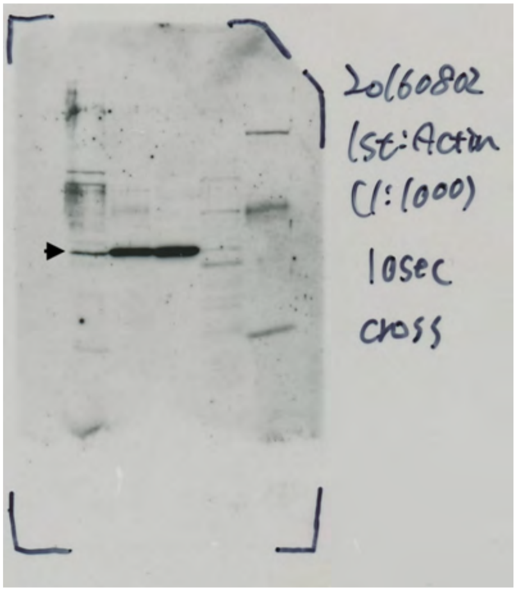

FigureS2_BmSOD1

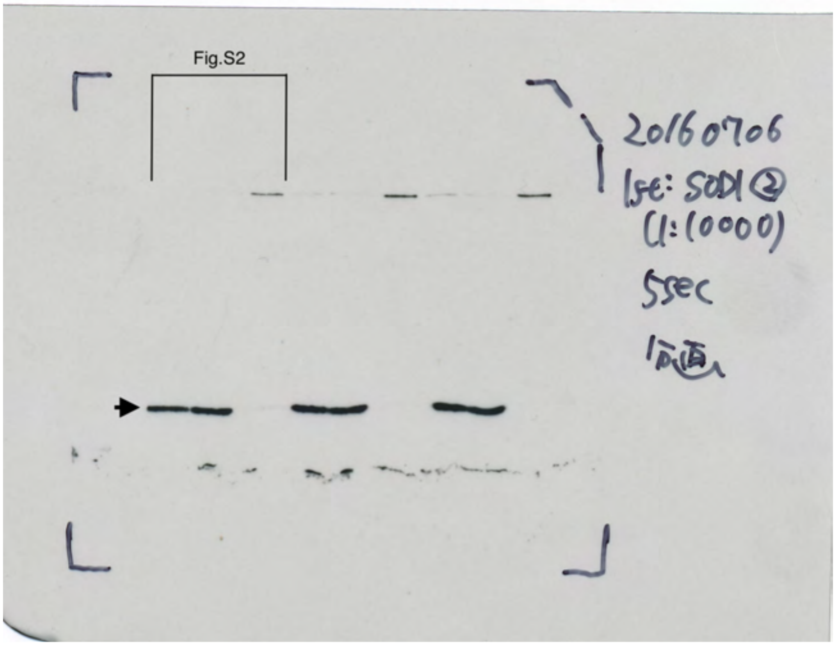

FigureS2_BmSOD2

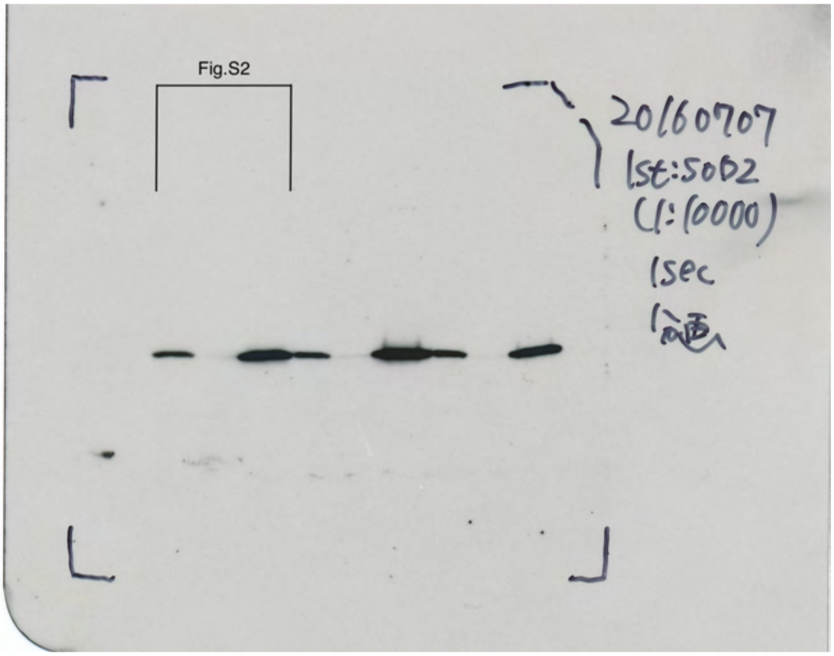

FigureS2_BmHsp60

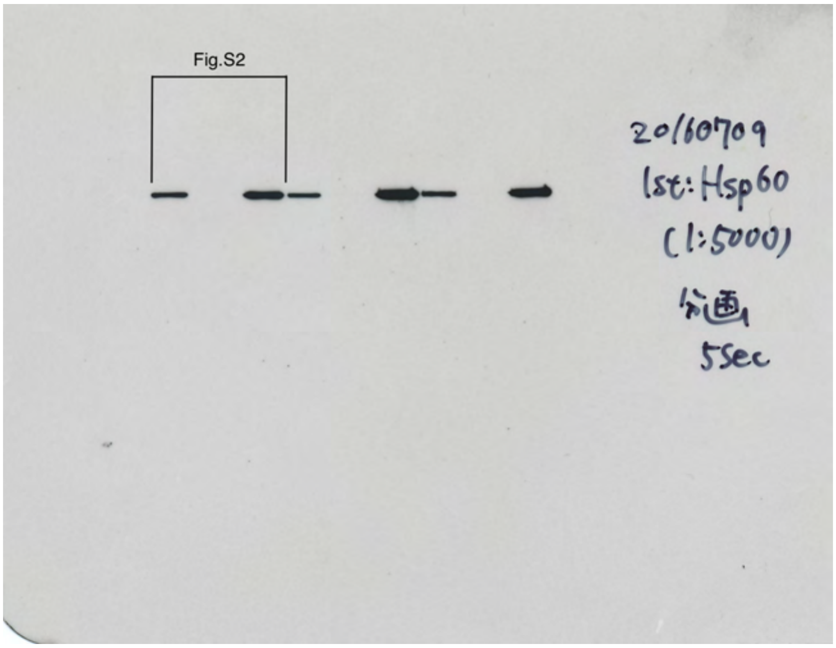

FigureS2_BmActin

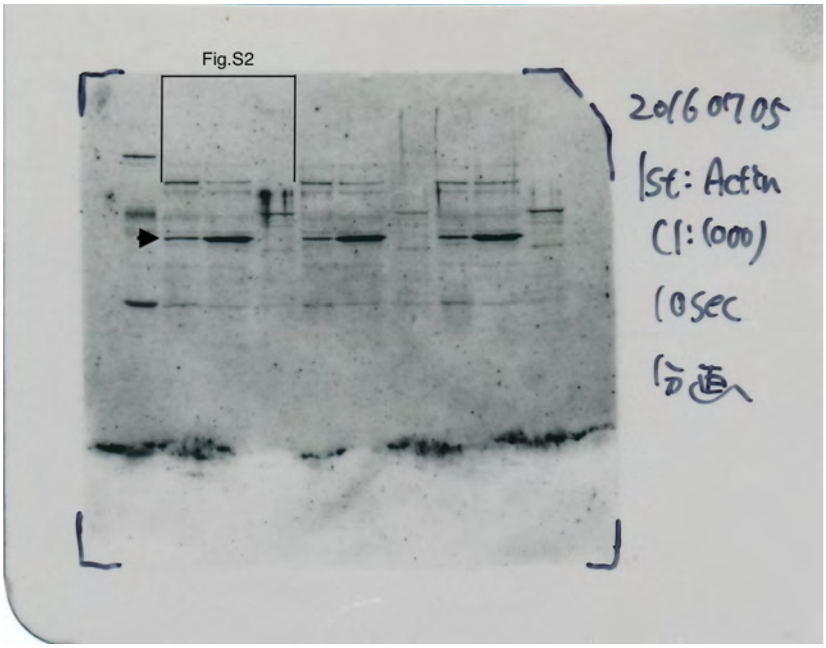

FigureS3_Mg-5th-larva_BmSOD1

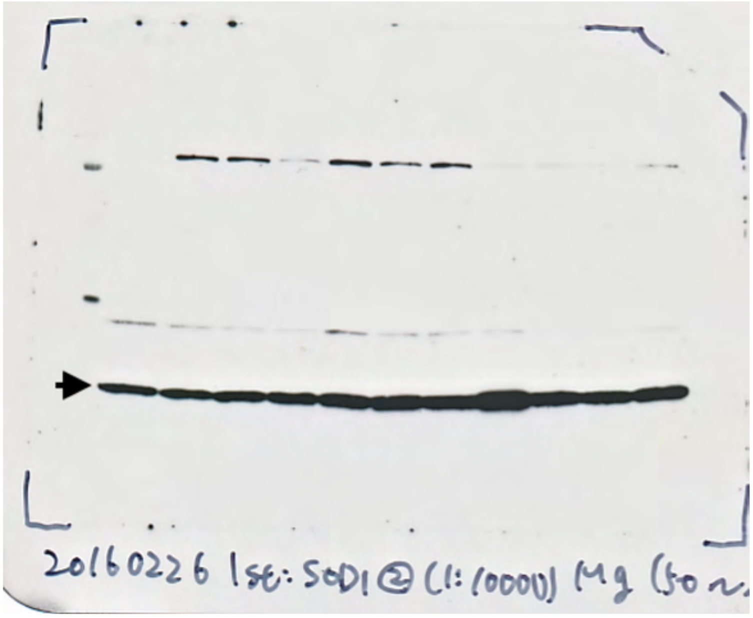

FigureS3_Mg-5th-larva_BmSOD2

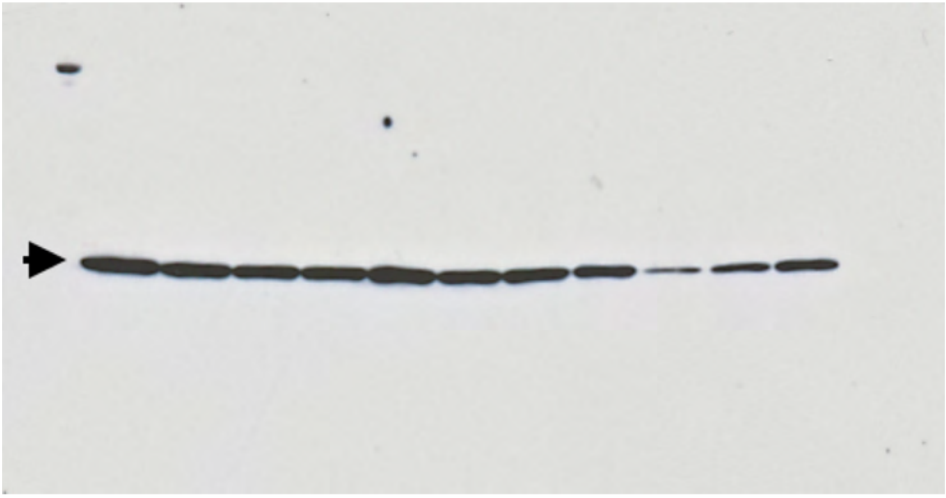

FigureS3_Mg-5th-larva_BmActin

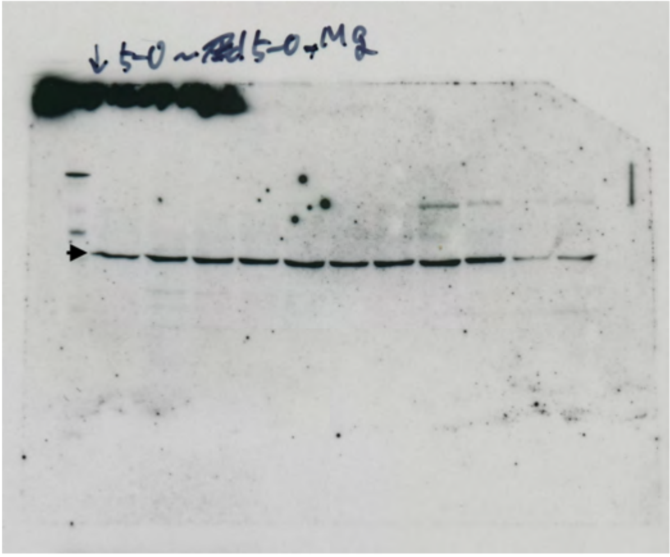

FigureS3_Mg-pupa-adult_BmSOD1

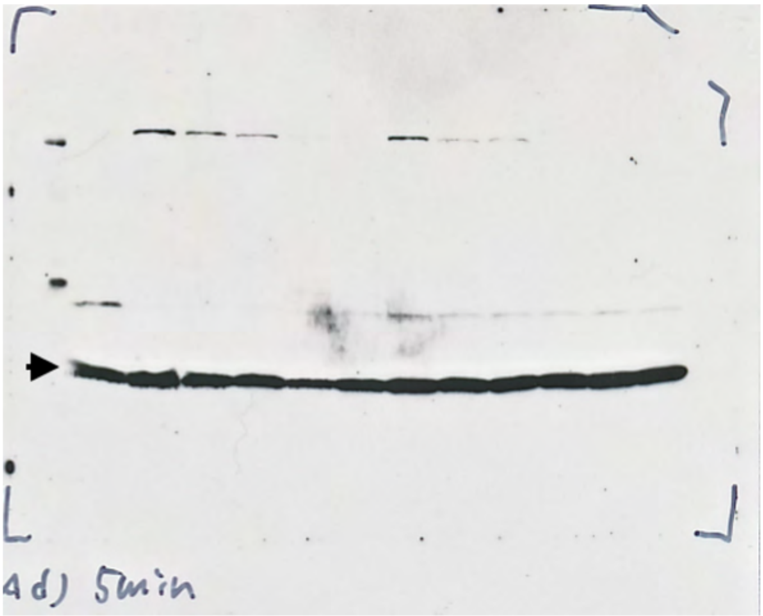

FigureS3_Mg-pupa-adult_BmSOD2

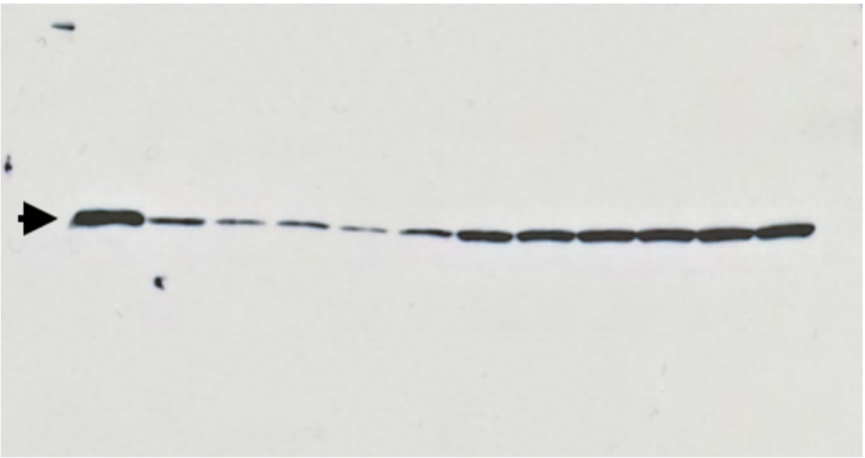

FigureS3_Mg-pupa-adult_BmActin

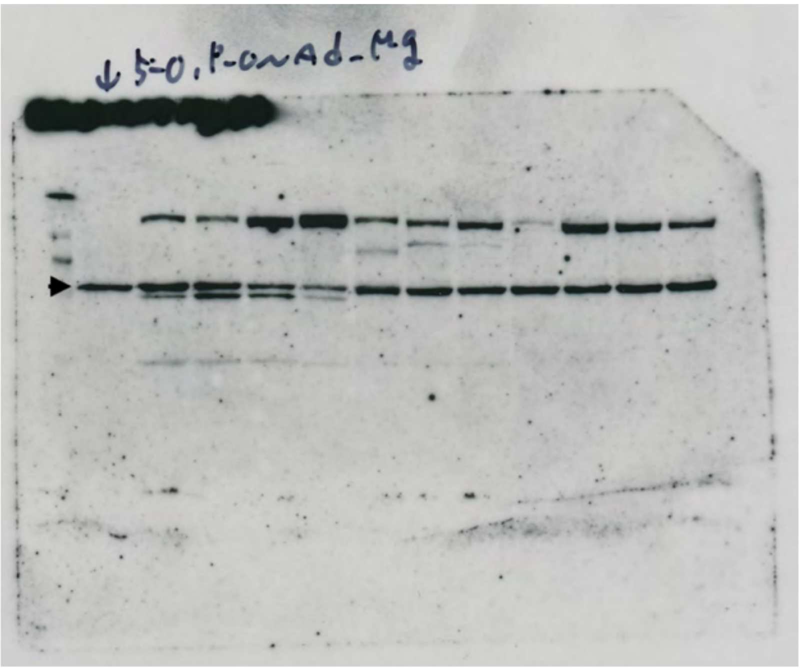

FigureS3_Mt-5th-larva_BmSOD1

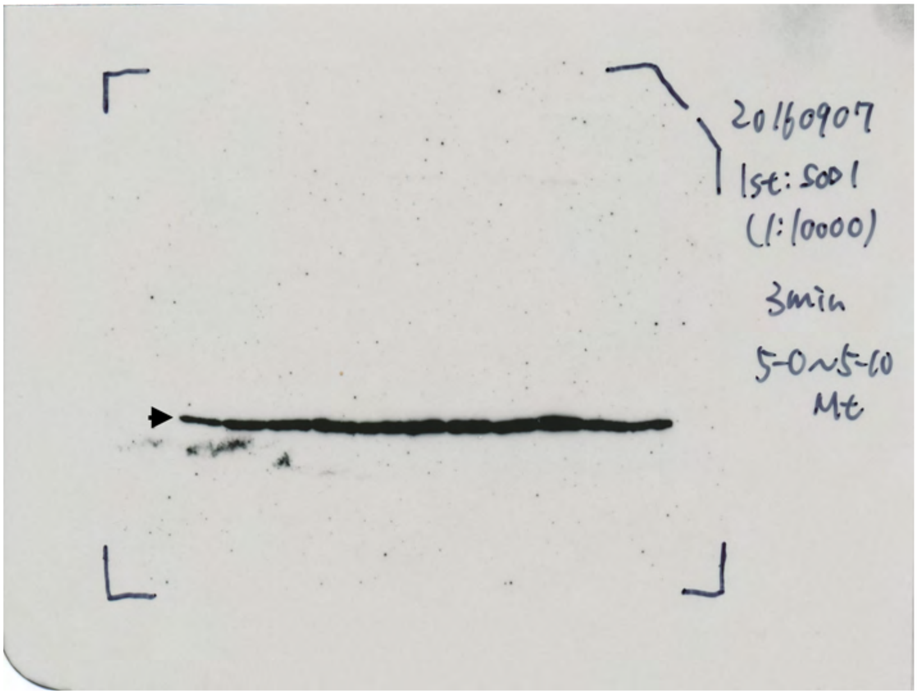

FigureS3_Mt-5th-larva_BmSOD2

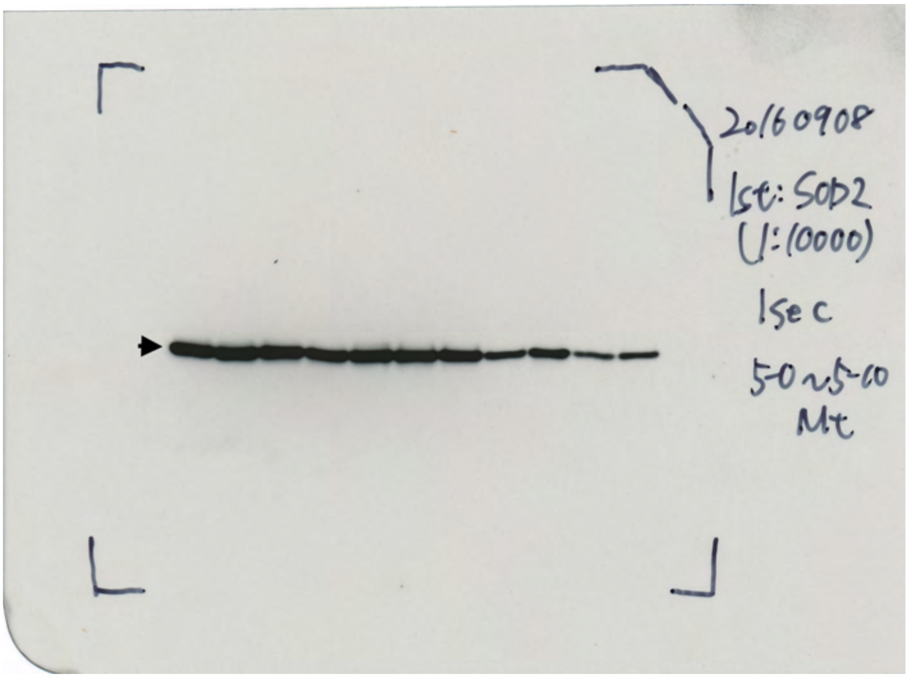

FigureS3_Mt-5th-larva_BmActin

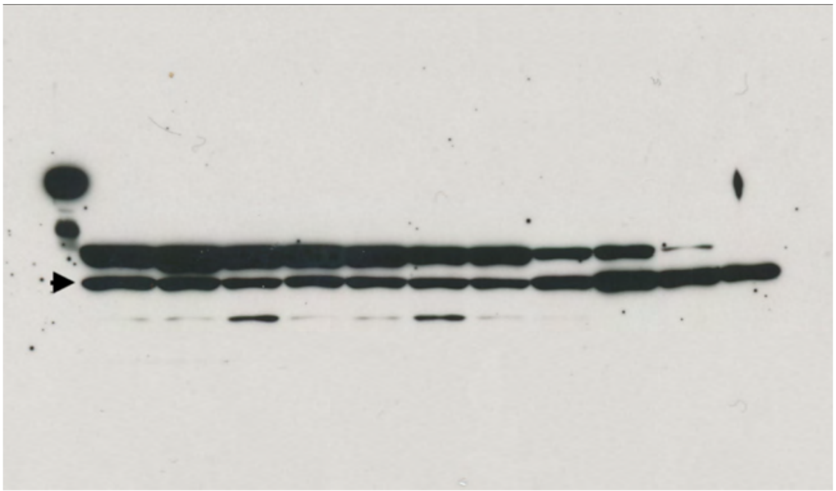

FigureS3_Mt-pupa-adult_BmSOD1

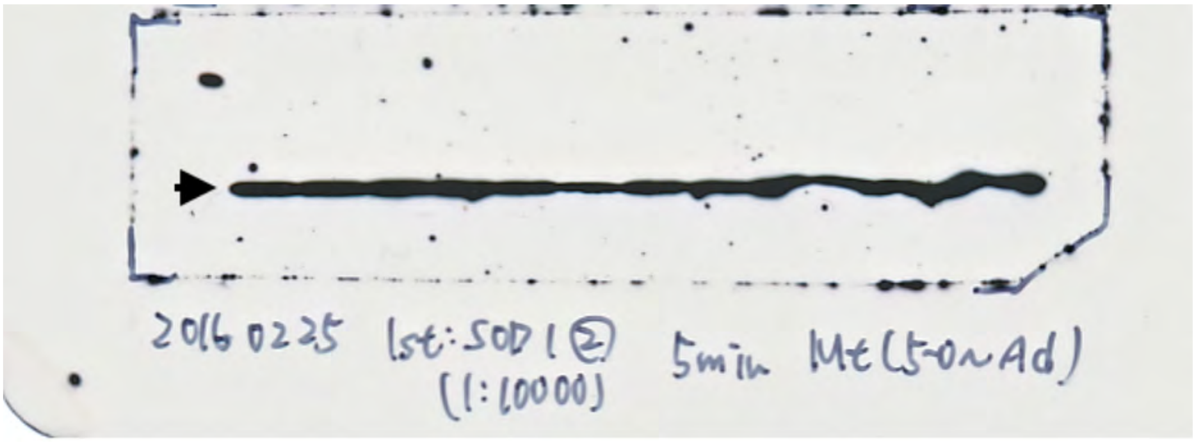

FigureS3_Mt-pupa-adult_BmSOD2

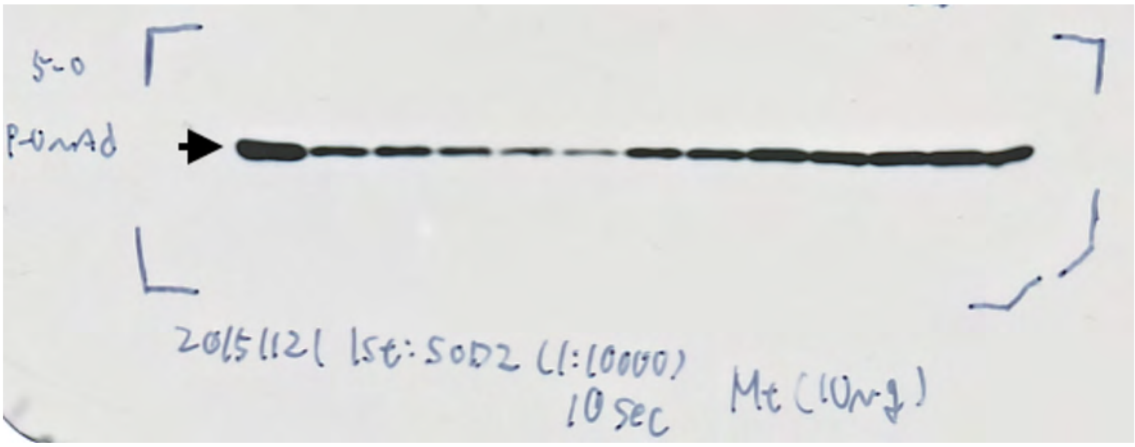

FigureS3_Mt-pupa-adult_BmActin

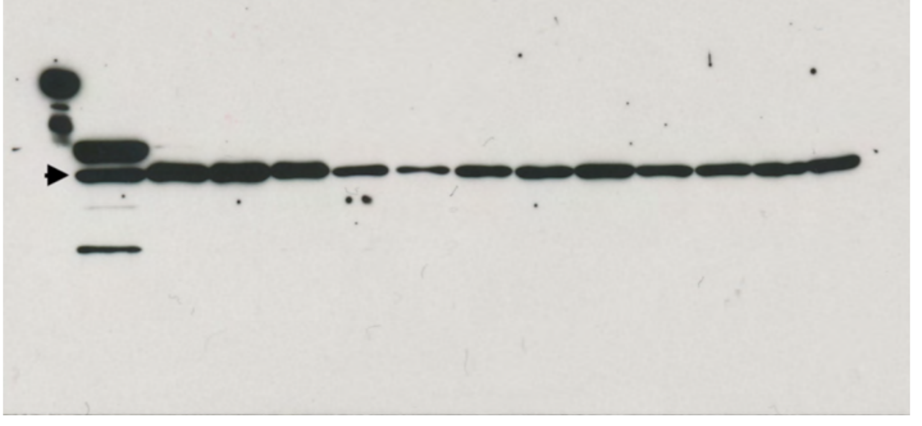

FigureS3_Ov-5th-larva_BmSOD1

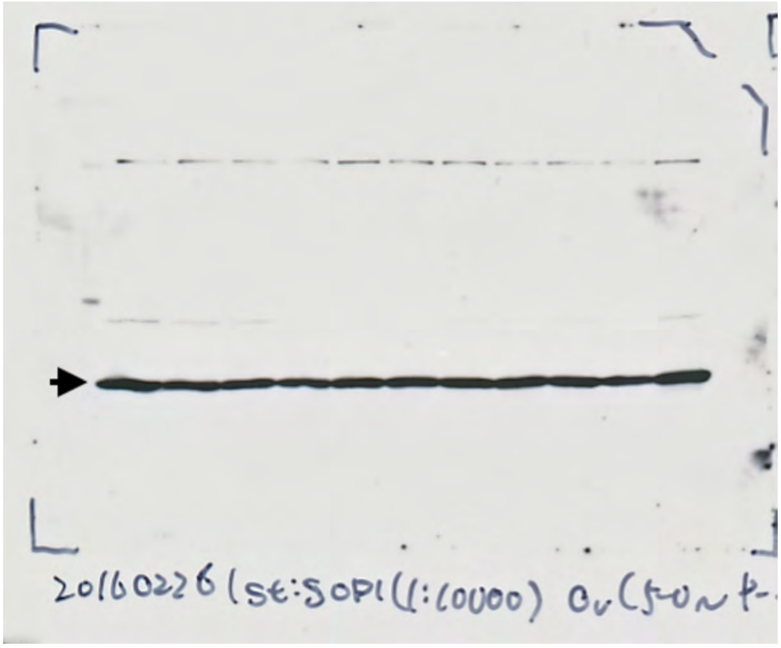

FigureS3_Ov-5th-larva_BmSOD2

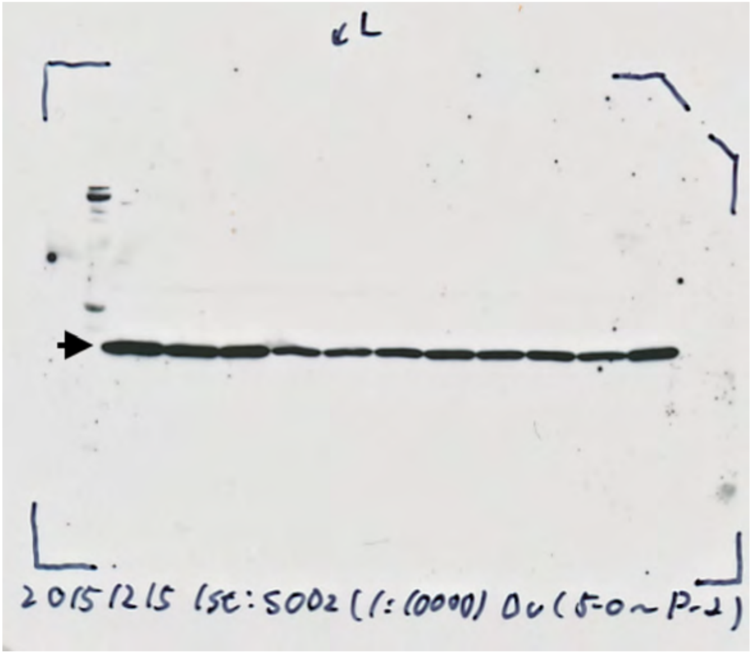

FigureS3_Ov-5th-larva_BmActin

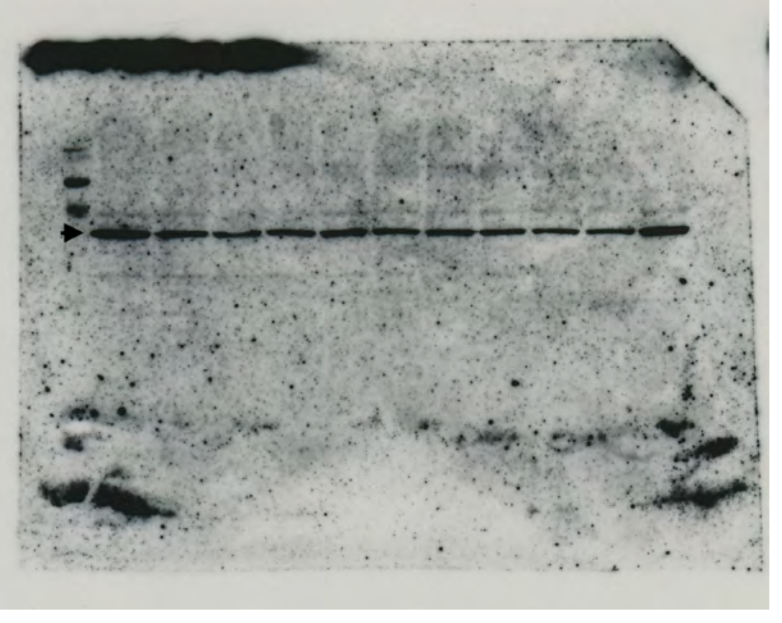

FigureS3_Ov-pupa_BmSOD1

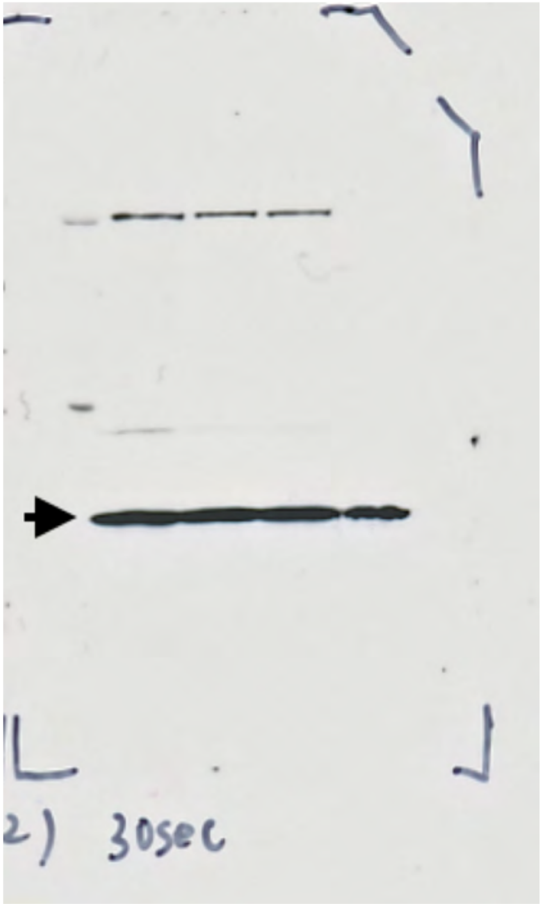

FigureS3_Ov-pupa_BmSOD2

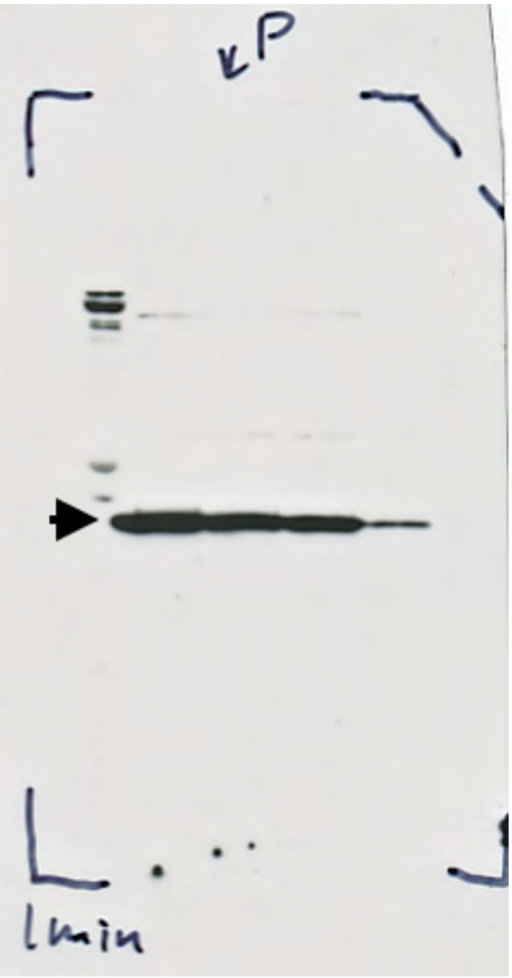

FigureS3_Ov-pupa_BmActin

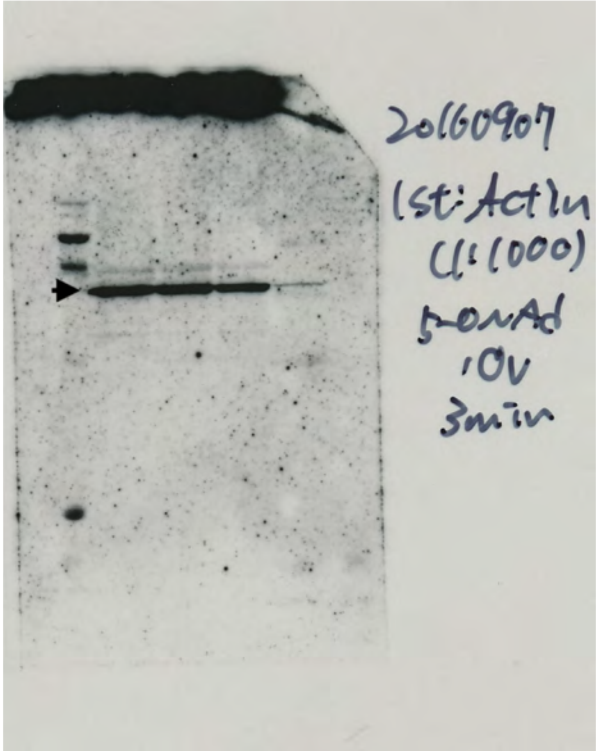

FigureS3_Tes-5th-larva_BmSOD1

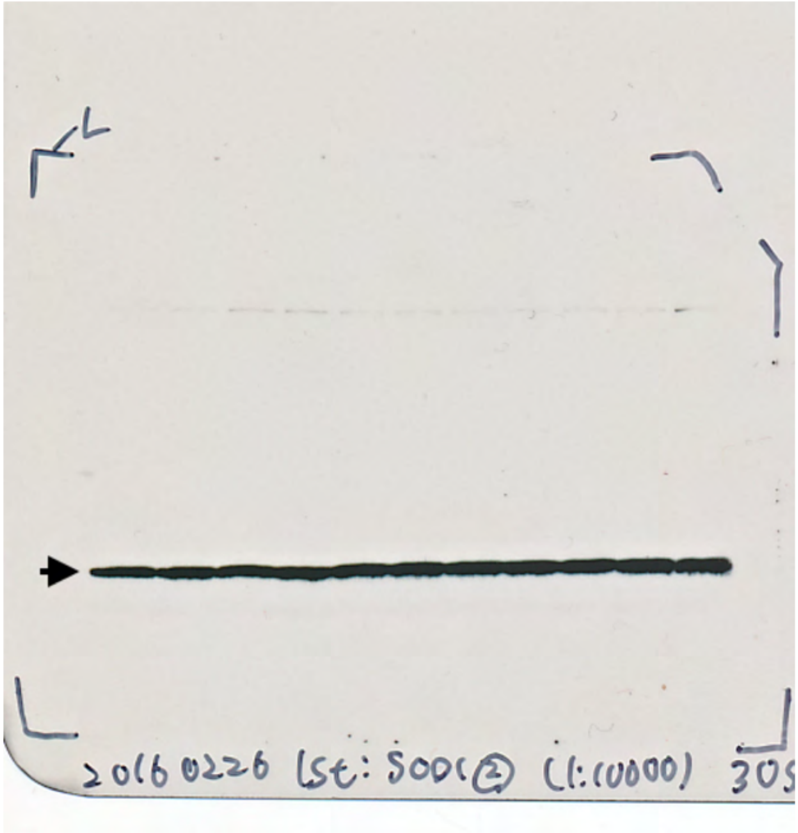

FigureS3_Tes-5th-larva_BmSOD2

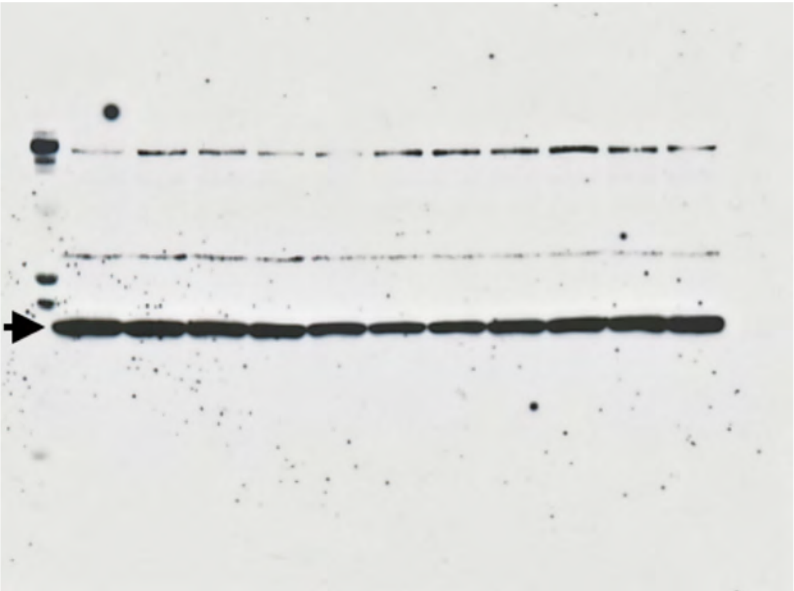

FigureS3_Tes-5th-larva_BmActin

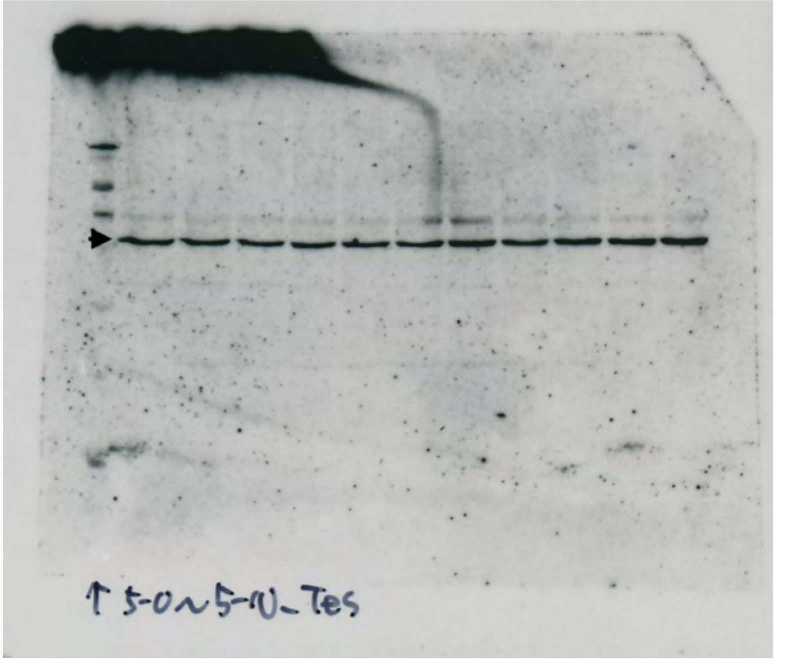

FigureS3_Tes-pupa-adult_BmSOD1

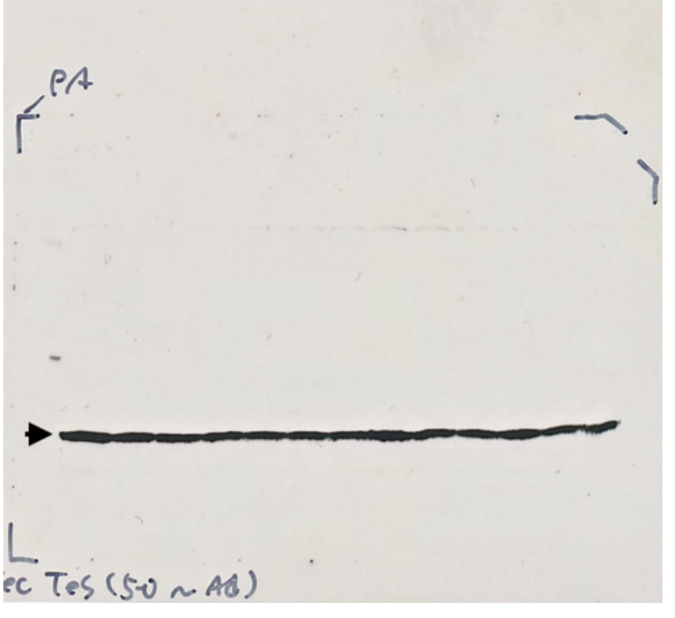

FigureS3_Tes-pupa-adult_BmSOD2

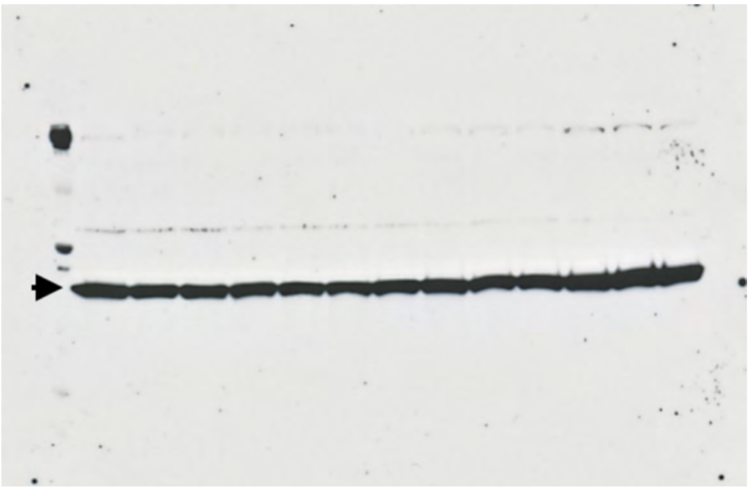

FigureS3_Tes-pupa-adult_BmActin

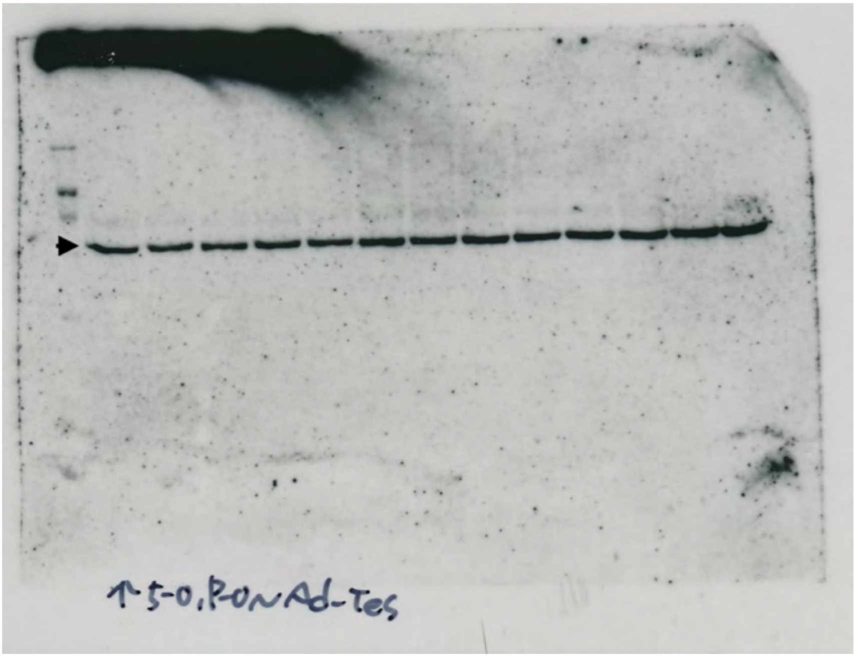

FigureS4-A_Fb-4th_BmSOD1

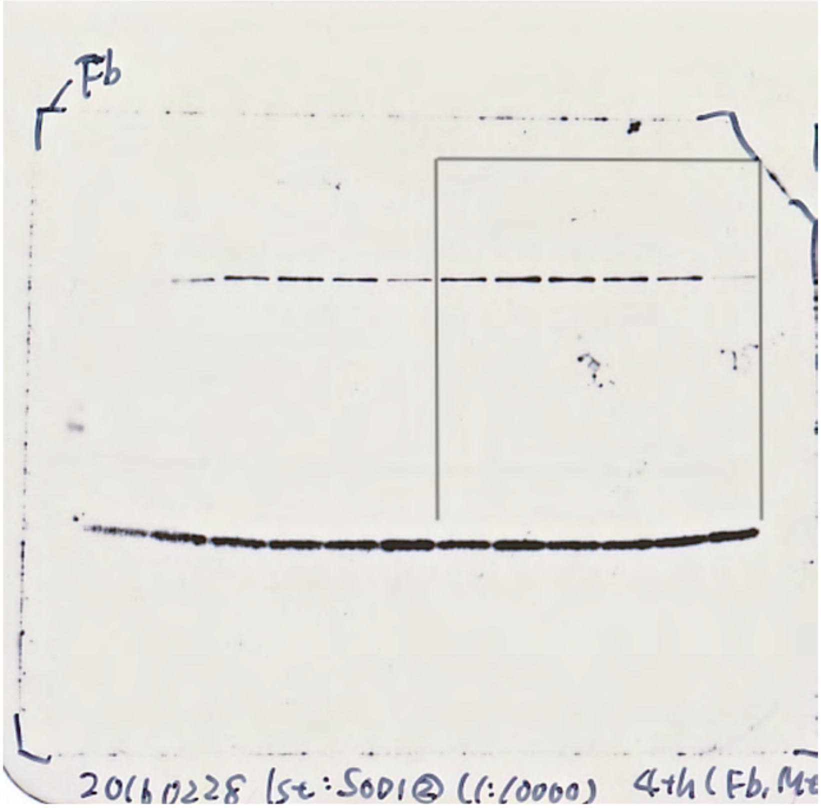

FigureS4-A_Fb-4th_BmSOD2

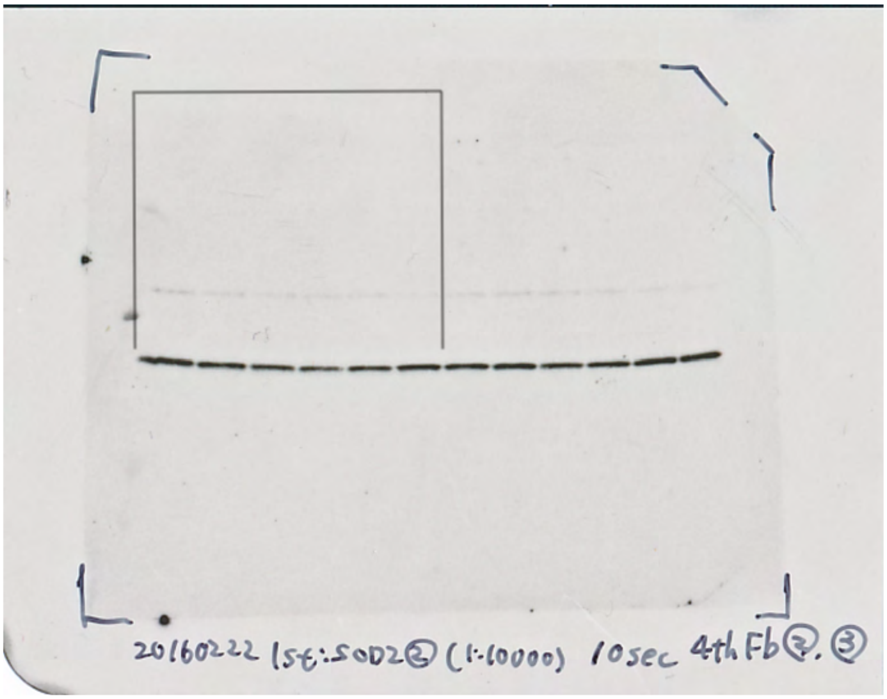

FigureS4-A_Fb-4th_BmActin

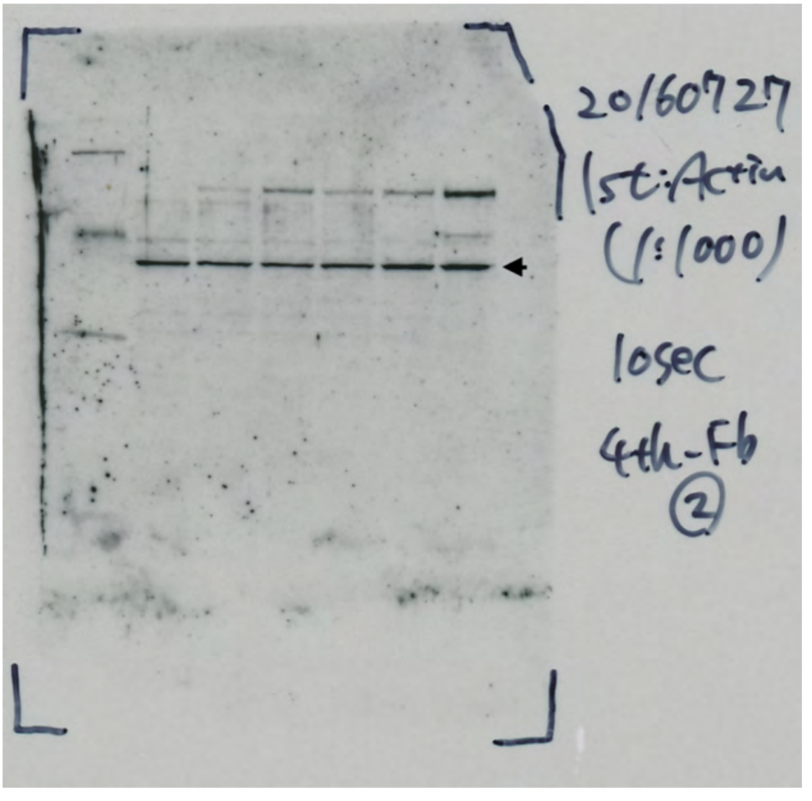

FigureS4-B_Mt-4th_BmSOD1

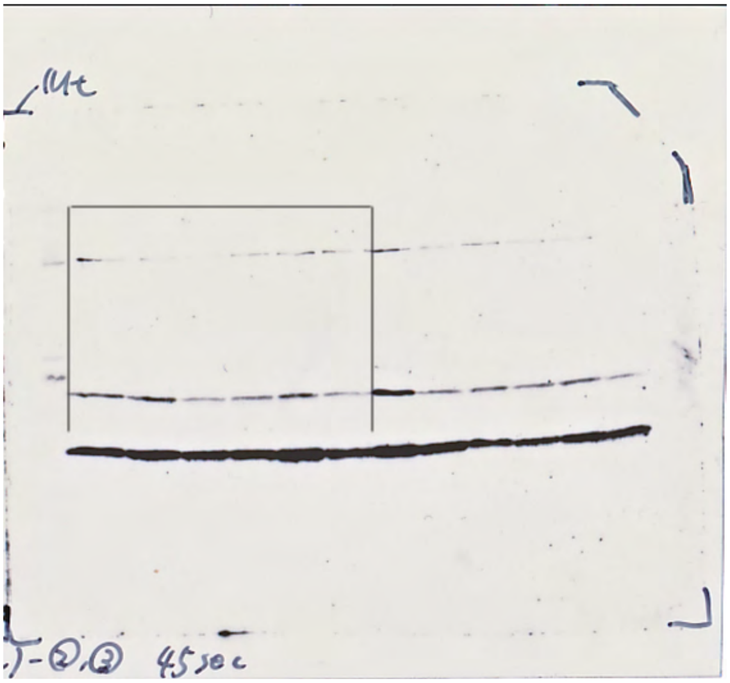

FigureS4-B_Mt-4th_BmSOD2

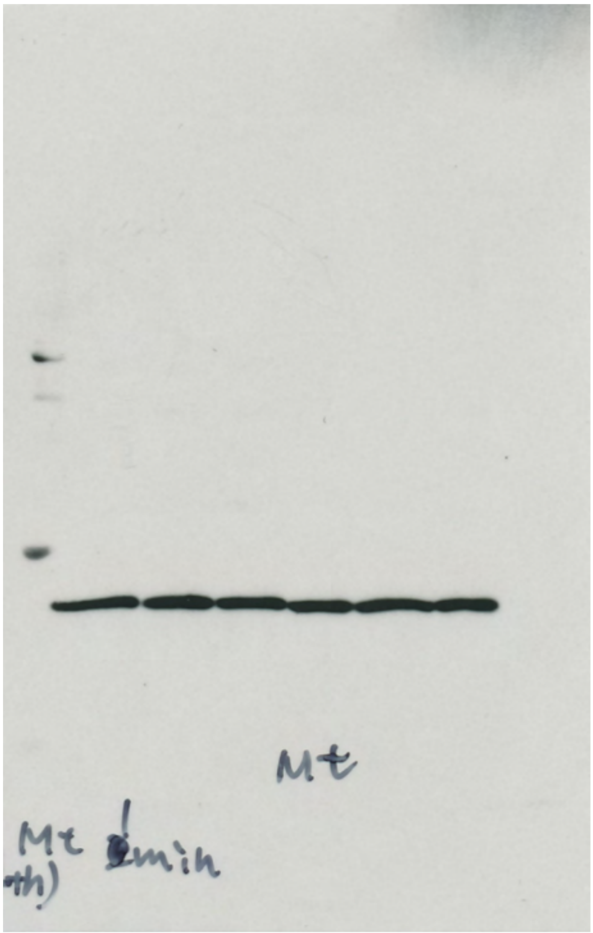

FigureS4-B_Mt-4th_BmActin

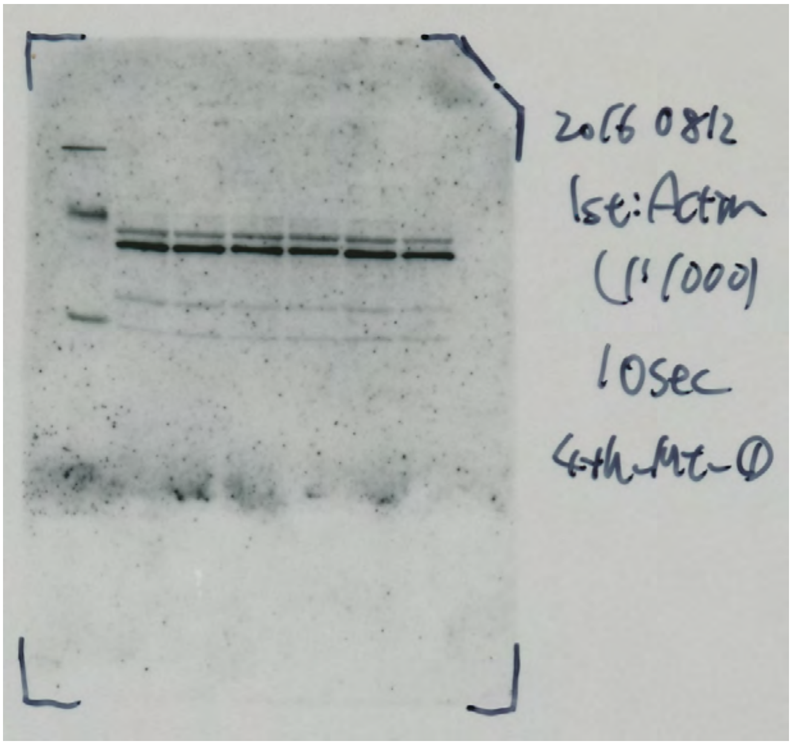

FigureS4-C_Mg-4th_BmSOD1,BmSOD2

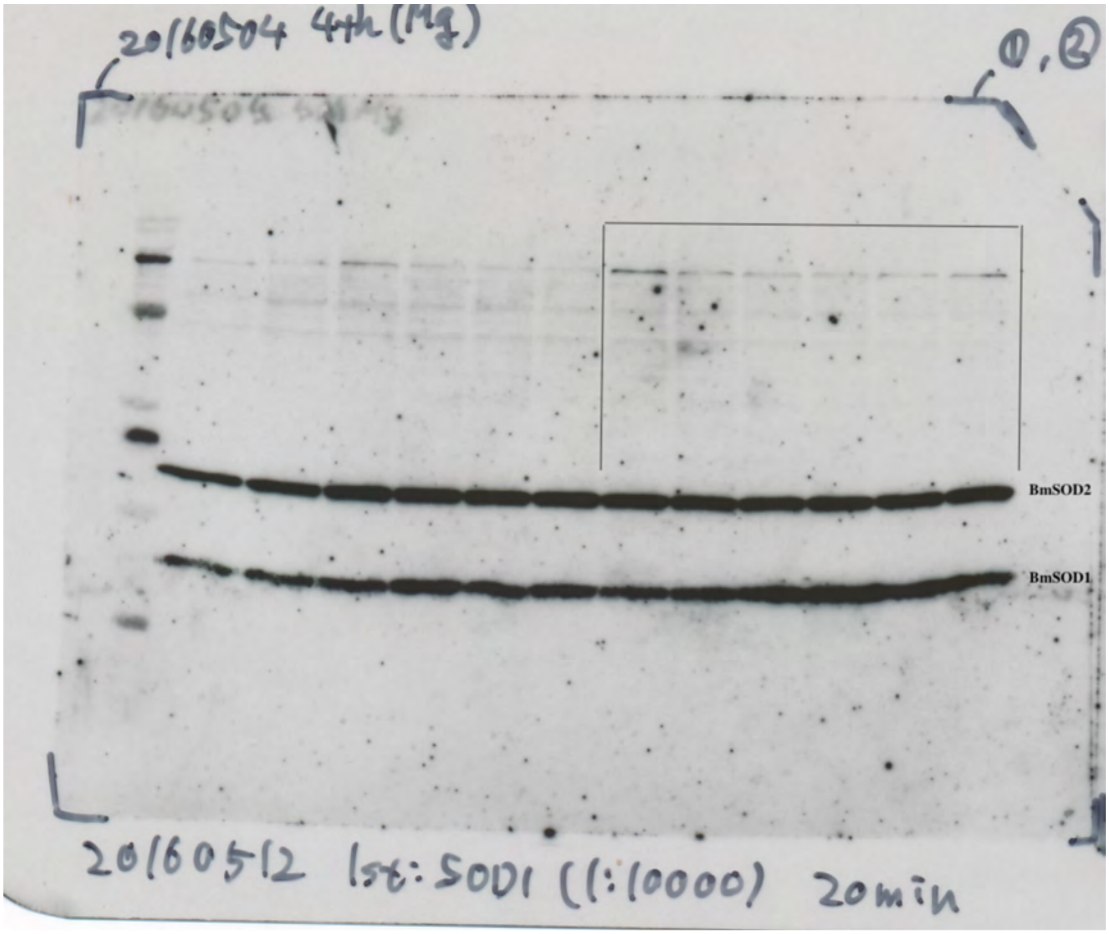

FigureS4-C_Mg-4th_BmActin

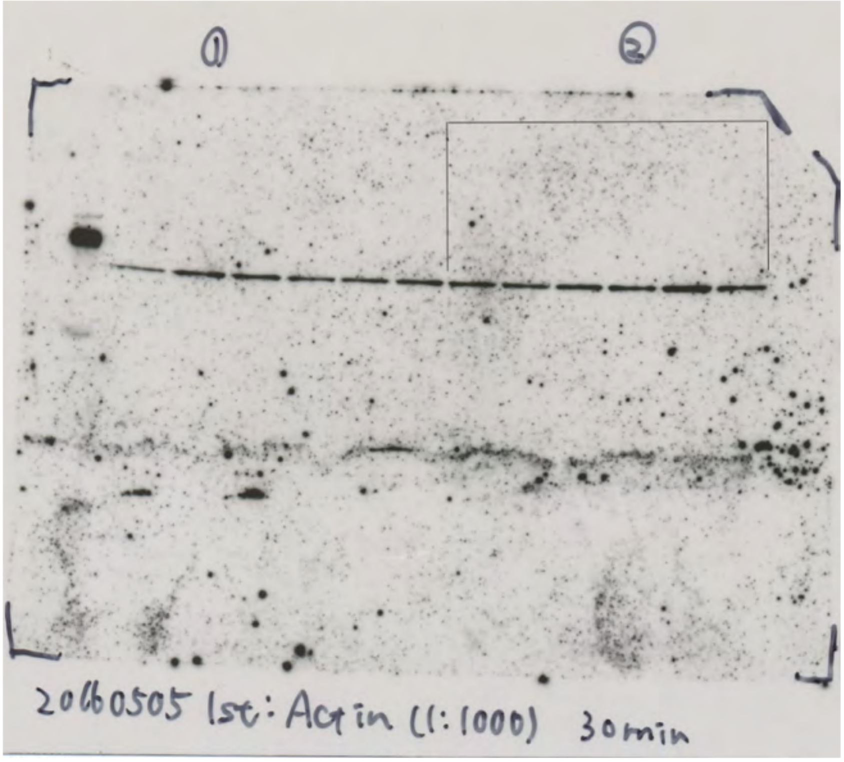

FigureS7_BmAtg1

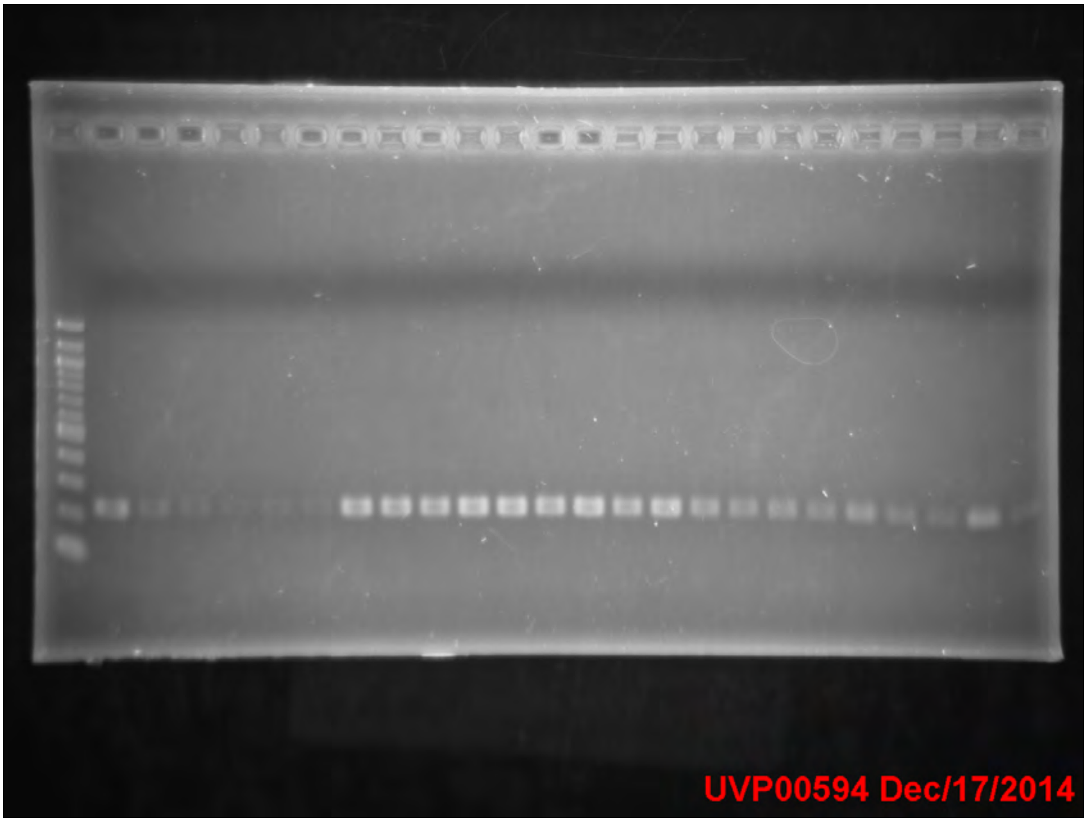

FigureS7_18s rRNA

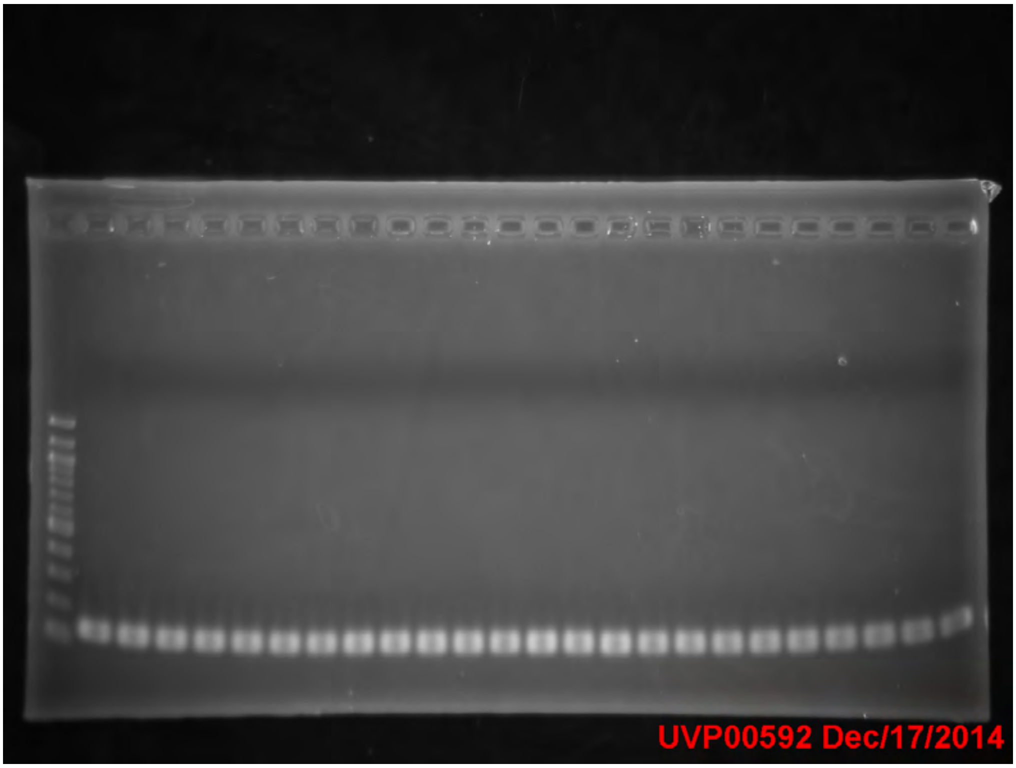

Supplement: Supplementary file 1 — Supplementary material [file 41598_2019_51163_MOESM1_ESM.pdf]
